# Evaluating data-driven methods for short-term forecasts of cumulative SARS-CoV2 cases

**DOI:** 10.1371/journal.pone.0252147

**Published:** 2021-05-21

**Authors:** Ghufran Ahmad, Furqan Ahmed, Muhammad Suhail Rizwan, Javed Muhammad, Syeda Hira Fatima, Aamer Ikram, Hajo Zeeb

**Affiliations:** 1 Department of International Business & Marketing, National University of Sciences and Technology (NUST), Islamabad, Pakistan; 2 Leibniz Institute for Prevention Research and Epidemiology, Bremen, Germany; 3 Health Sciences Bremen, University of Bremen, Bremen, Germany; 4 Department of Finance & Investment, National University of Sciences and Technology (NUST), Islamabad, Pakistan; 5 Department of Microbiology, University of Haripur, Haripur, Pakistan; 6 School of Public Health, University of Adelaide, Adelaide, Australia; 7 National Institute of Health (NIH), Islamabad, Pakistan; South China University of Technology, CHINA

## Abstract

**Background:**

The WHO announced the epidemic of SARS-CoV2 as a public health emergency of international concern on 30th January 2020. To date, it has spread to more than 200 countries and has been declared a global pandemic. For appropriate preparedness, containment, and mitigation response, the stakeholders and policymakers require prior guidance on the propagation of SARS-CoV2.

**Methodology:**

This study aims to provide such guidance by forecasting the cumulative COVID-19 cases up to 4 weeks ahead for 187 countries, using four data-driven methodologies; autoregressive integrated moving average (*ARIMA*), exponential smoothing model (*ETS*), and random walk forecasts (*RWF*) with and without drift. For these forecasts, we evaluate the accuracy and systematic errors using the Mean Absolute Percentage Error (*MAPE*) and Mean Absolute Error (*MAE*), respectively.

**Findings:**

The results show that the *ARIMA* and *ETS* methods outperform the other two forecasting methods. Additionally, using these forecasts, we generate heat maps to provide a pictorial representation of the countries at risk of having an increase in the cases in the coming 4 weeks of February 2021.

**Conclusion:**

Due to limited data availability during the ongoing pandemic, less data-hungry short-term forecasting models, like *ARIMA* and *ETS*, can help in anticipating the future outbreaks of SARS-CoV2.

## Introduction

Severe Acute Respiratory Syndrome Coronavirus-2 (SARS-CoV2) is a zoonotic virus belonging to the betacoronavirus group of the coronaviridae family which also includes SARS-CoV and MERS. These viruses are known to cause severe acute respiratory diseases in humans [1]. The first confirmed case of SARS-CoV2 emerged in December 2019 in Wuhan, China. The WHO announced the epidemic of SARS-CoV2 as a public health emergency of international concern on 30th January 2020 due to the high human to human transmission rate and absence of any treatment or vaccine [2]. To date, it has spread to more than 200 countries and has been declared as a global pandemic [3]. SARS-CoV2 transmits through respiratory droplets and has a binding capacity, through spike proteins, to angiotensin-converting enzyme 2 (ACE2) receptors in the human respiratory system [3]. Clinical symptoms of SARS-CoV2 include cough, fever, shortness of breath, and—in severe cases—pneumonia and multiple organ failure [3]. SARS-CoV2 has an incubation period of 1–14 days and a substantial proportion of the infected persons appear to be asymptomatic. Moreover, these individuals are highly infectious before the onset of symptoms, which makes it a challenge to diagnose, contain, and control transmissions [1, 4]. Initially, the mean basic reproduction number (*R*_0_) of SARS-CoV2 ranged from 1.4 to 6.49, while some studies highlighted that the *R*_0_ stabilized around 2–3 leading to an exponential increase in the number of cases [3, 5]. However, relying solely on *R*_0_ to formulate policies could be misleading as there may be a possibility of a COVID-19 outbreak even when *R*_0_ is less than one [6].

Globally, many public and private enterprises are exploring treatment options and are in the process of vaccine development. However, vaccines have to go through a robust and usually time-consuming process of clinical trials owing to the paradigms of human safety, health, and bioethics [7]. Vaccine rollout has commenced in several countries but due to production limitations, maintaining the cold chain, and vaccine hesitancy, the trend of the number of administered doses is sluggish [7, 8]. Globally, countries have implemented various interventions in an attempt to limit transmissions and curtail the number of deaths caused by COVID-19. These interventions include social distancing, the closing of public places, academic institutes and schools, travel restrictions, quarantine for the infected, and—in some cases—curfew or lockdown [9]. However, issues in the health security infrastructure, disease surveillance, health systems, and limited availability of health professionals makes it a challenge for containing, mitigating, and rolling out the vaccine for SARS-CoV2 [9].

Considering these concerns, for appropriate preparedness, containment, and mitigation response, the stakeholders and policymakers require prior guidance on the propagation of SARS-CoV2. This study aims to provide such guidance by forecasting the cumulative COVID-19 cases up to 4 weeks ahead and ascertain their accuracy using the Mean Absolute Percentage Error and Mean Percentage Error.

## Data sources

The daily level data for the cumulative COVID-19 cases, for 187 countries, and at the aggregated level for the entire world, was acquired from “Our World in Data”–a combined effort of the researchers at the University of Oxford and the Global Change Data Lab—which relies on the European Centre for Disease Prevention and Control (ECDC) for data collection [10, 11]. Our sample period starts from the day of the first reported case for each country till 1^st^ February 2021. The statistical analysis of this paper was performed using R 4.0.3.

Our variable of interest, for the forecasting analysis, is the cumulative COVID-19 cases at the daily level. The descriptive statistics of the cumulative COVID-19 cases—number of observations, standard deviation, minimum, and maximum—are presented in Table 1. For brevity, Table 1 only provides the descriptive statistics for the 29 countries with the highest cumulative COVID-19 cases as of 1^st^ February 2021 and at the aggregated level for the entire world. The descriptive statistics for the entire sample are provided in S1 Table.

**Table 1:** Descriptive statistics of the 29 countries with the highest cumulative COVID-19 cases and at the aggregated level of the entire world.

Country	Observations	Standard Deviation	Minimum	Maximum
Argentina	336	654867.2	1	1933853
Belgium	364	245428.4	1	711417
Brazil	342	2897244	1	9229322
Canada	373	206854.9	1	788186
Chile	345	229784.1	2	730888
Colombia	333	644995.2	1	2104506
Czechia	338	295867.7	3	987329
France	375	1017765	2	3260308
Germany	372	613919.7	1	2232327
India	369	4124427	1	10766245
Indonesia	337	292201.6	2	1089308
Iran	349	421107.1	2	1424596
Iraq	344	237033.7	1	620620
Israel	347	179182.7	1	652246
Italy	368	755731.6	2	2560957
Mexico	340	536228.6	1	1869708
Netherlands	341	298779.7	1	995300
Peru	333	390101.4	1	1138239
Poland	335	503521.2	1	1515889
Portugal	337	172793.4	2	726321
Romania	342	235456	1	730056
Russia	368	1092717	2	3825739
South Africa	334	418621.3	1	1456309
Spain	367	743438.9	1	2822805
Sweden	364	157663.7	1	576606
Turkey	328	755429.5	1	2485182
UK	368	983256	2	3846851
USA	377	7199436	1	26321120
Ukraine	336	402486.9	1	1263833
World	377	30371381	557	103422636

## Ethics

No ethics approval was required for the study as secondary data analysis was performed on the publicly available COVID-19 dataset.

## Forecasting methodology and evaluation

To forecast the cumulative COVID-19 cases, we use four different forecasting methods. Three of the forecasts are based on the autoregressive integrated moving average process which is usually denoted as *ARIMA*(*p*, *d*, *q*) where *p* is the order of the autoregressive model, *d* is the degree of differencing, and *q* is the order of the moving average model. The *ARIMA* model has been used for forecasting and assessing seasonality in infectious disease outbreaks [12–15].

The *ARIMA* model is a generalization of the autoregressive moving average (*ARMA*) model with an ability to address the potential non-stationarity of the variable of interest. To test for stationarity of the cumulative COVID-19 cases, we used the Augmented Dickey-Fuller (*ADF*) and Phillips-Perron (*PP*) unit root tests. The null hypothesis of these tests is that the variable contains a unit root, hence non-stationary, whereas the alternative is that the time series variable was generated by a stationary process. Table 2 reports the p-values, for the unit root tests, for the 29 countries with the highest cumulative COVID-19 cases and at the aggregated level for the entire world. S2 Table provides the results of the unit root tests for the 187 countries. The test results suggest non-stationarity which justifies the use of the *ARIMA* model.

**Table 2:** Results of the unit root tests for the 29 countries with the highest cumulative COVID-19 cases and at the aggregated level of the entire world.

Country	*ADF*^a^	*PP*^b^
Argentina	1.00	1.00
Belgium	1.00	1.00
Brazil	1.00	1.00
Canada	1.00	1.00
Chile	1.00	1.00
Colombia	1.00	1.00
Czechia	1.00	1.00
France	1.00	1.00
Germany	1.00	1.00
India	1.00	1.00
Indonesia	1.00	1.00
Iran	1.00	1.00
Iraq	1.00	1.00
Israel	1.00	1.00
Italy	1.00	1.00
Mexico	1.00	1.00
Netherlands	1.00	1.00
Peru	1.00	0.99
Poland	1.00	1.00
Portugal	1.00	1.00
Romania	1.00	1.00
Russia	1.00	1.00
South Africa	1.00	1.00
Spain	1.00	1.00
Sweden	1.00	1.00
Turkey	1.00	1.00
UK	1.00	1.00
USA	1.00	1.00
Ukraine	1.00	1.00
World	1.00	1.00

^a^ The p-values of the Augmented Dickey-Fuller unit root test.

^b^ The p-values of the Phillips-Perron unit root test.

Let *X*_*t*_ denote the cumulative COVID-19 cases on the *t*^th^ day for the country being analyzed. Then, *ARIMA*(*p*, *d*, *q*) equation can be given as follows [16]:

$$D^{d}\cdot X_{t}^{\;}=\alpha +\sum_{i=1}^{p}\beta_{i}(D^{d}\cdot X_{t-i}^{\;})+\sum_{j=1}^{q}\gamma_{i}\varepsilon_{t-i}^{\;}+\varepsilon_{t}$$ (1)

In Eq (1), *D* is the difference operator, *α* is the constant term, *β*’s and *γ*’s are the coefficients of the autoregressive and the moving average component of the *ARIMA* model, respectively, and *ε* is the error term which is assumed to be independently and identically distributed from a normal distribution with zero mean. Eq (1) shows that the *AR* component allows the variable to be determined based on its prior values whereas the *MA* component shows that the error term is a linear combination of the current and prior values of *ε*. The latter accounts for the autocorrelation in the variable of interest.

A particularly naïve attempt is to fit *ARIMA*(0,1,0) which is commonly referred to as random walk and its forecasts are termed as random walk forecasts (*RWF*). We generate the *RWF* with and without drift for the variable of interest.

A more systematic approach for fitting the *ARIMA* model follows these steps [17]:

1. To ensure stationarity, the differencing order (*d*) is selected by using the Kwiatkowski-Phillips-Schmidt-Shin test [18].
2. The lags, *p* and *q*, are determined by using the Akaike Information Criterion corrected for small sample sizes.

Aside from the *ARIMA* model, we also used the exponential smoothing method (*ETS*) for generating forecasts. *ETS* is a forecasting method for univariate data which deals with the systematic trend, seasonality, and can be used as an alternative to the *ARIMA* models [19].

To evaluate the performance of forecasts, the data is divided into two mutually exclusive sets, the training and test sets. The training set is used to fit the model (without using any data from the test set) whereas the test set is kept for evaluating the forecast accuracy. We use a variant of the time series cross-validation which is a more sophisticated version of the usual training-test set methodology [16]. In this method, there is a series of test sets, and each test set is accompanied by a corresponding training set consisting of observations before the test set. Therefore, a series of training-test sets are constructed, and for each training-test set forecast accuracy is determined. This method is more sophisticated than the usual training-test set methodology because it allows more comparisons of the forecasted and actual data values.

The time-series cross-validation method is also referred to as evaluation on a rolling forecasting origin because the origin of the test set is rolled forward in time. In simpler words:

1. An origin for the first test set is selected.
2. Forecasts are determined for the test set using the corresponding training set.
3. The origin is rolled forward by one period generating a new training-test set for which forecasts can be evaluated, and so on.

In this study, we take the 45^th^ day—since the first reported case in the country—as the origin which is then rolled forward one day at a time. The variation in our methodology is that, instead of taking each of the test set as a single observation, we take four different test sets for each training set: 1 week, 2 weeks, 3 weeks, and 4 weeks into the future. This allows us to ascertain the accuracy of the forecasting method up to 4 weeks ahead for each training set. Therefore, we include countries with at least 73 (45+28) observations to ensure that there is at least one available test set for the 4 weeks ahead forecasts for each country included for the forecast evaluation.

Suppose the country under consideration has data available for *t* ∈ {1,2,⋯, *T*}. The following steps explain the methodology:

1. Use the data available till *t* = 45, and forecast the values of *X*_*t*+*τ*_ for *τ* ∈ {1,2,⋯, 28}, i.e., obtain the forecasts for the next 28 days or 4 weeks.
2. Construct 1 week, 2 weeks, 3 weeks, and 4 weeks ahead forecasts using the forecasted values till *t* + 7, *t* + 14, *t* + 21, and *t* + 28, respectively.
3. Increase the data sample by one day, i.e., take the data till (*t* + 1)^th^ day and obtain 28-day ahead forecasts, and repeat this process until we reach the end of the data, i.e., we reach the *T*^th^ day.

There are several methods to determine the accuracy of the forecasted values. We used the Mean Absolute Percentage Error (*MAPE*) for this purpose which is defined as follows [16]:

$$MAPE=100\times \frac{1}{n}\sum_{i=1}^{n}\left|\frac{A_{i}-F_{i}}{A_{i}}\right|$$ (2)

In Eq (2), *A*_*i*_ and *F*_*i*_ denote the actual and forecasted values, respectively, and *n* is the number of forecasted values for which a corresponding actual data value exists. It should be clear that forecasting accuracy increases as *MAPE* becomes closer to zero. Since the forecasted variable of this study is the cumulative COVID-19 cases, *MAPE* represents the forecasting error as the percentage of cumulative COVID-19 cases. Based on our methodology, there is a series of training-test sets, and *MAPE* can be determined for each of these. Therefore, the forecasting accuracy is calculated by averaging *MAPE* over the series of the training-test sets [16].

We also estimate the Mean Percentage Error (*MPE*) which is defined as follows [20]:

$$MPE=100\times \frac{1}{n}\sum_{i=1}^{n}\frac{A_{i}-F_{i}}{A_{i}}$$ (3)

Since *MAPE* uses the absolute values of the forecasting errors, it is unable to determine whether the forecasting model is systematically under or over-predicting. In this regard, *MPE* can prove useful as it does not use the absolute values of the forecasting errors [20].

## Results

This section presents our results; the forecasting accuracy of the four forecasting methodologies, the forecasted values, and the heat maps.

### Forecasting evaluation

**Fig 1:**
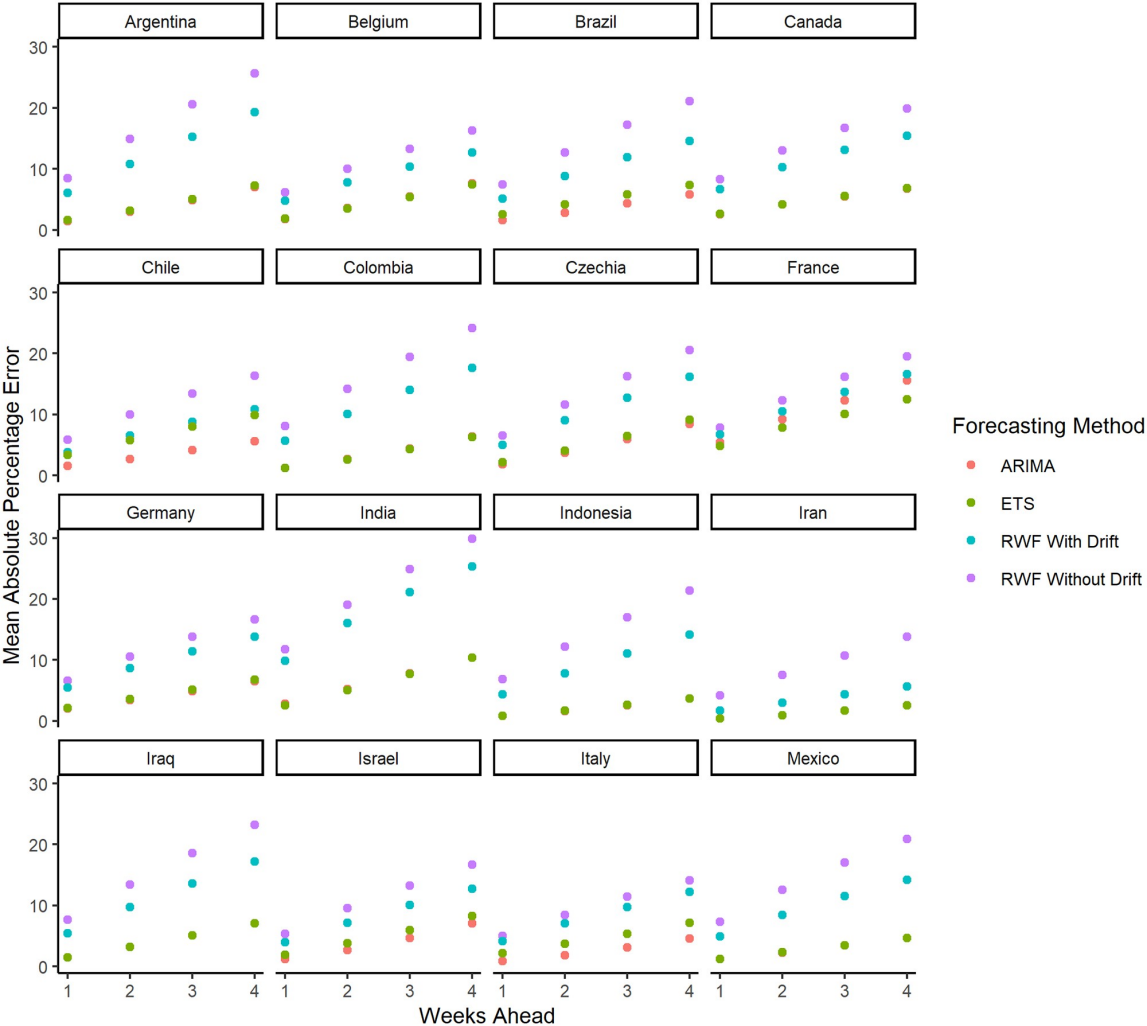
The *MAPE* for the forecasted values for 16 of the 29 countries with the highest cumulative COVID-19 cases.

**Fig 2:**
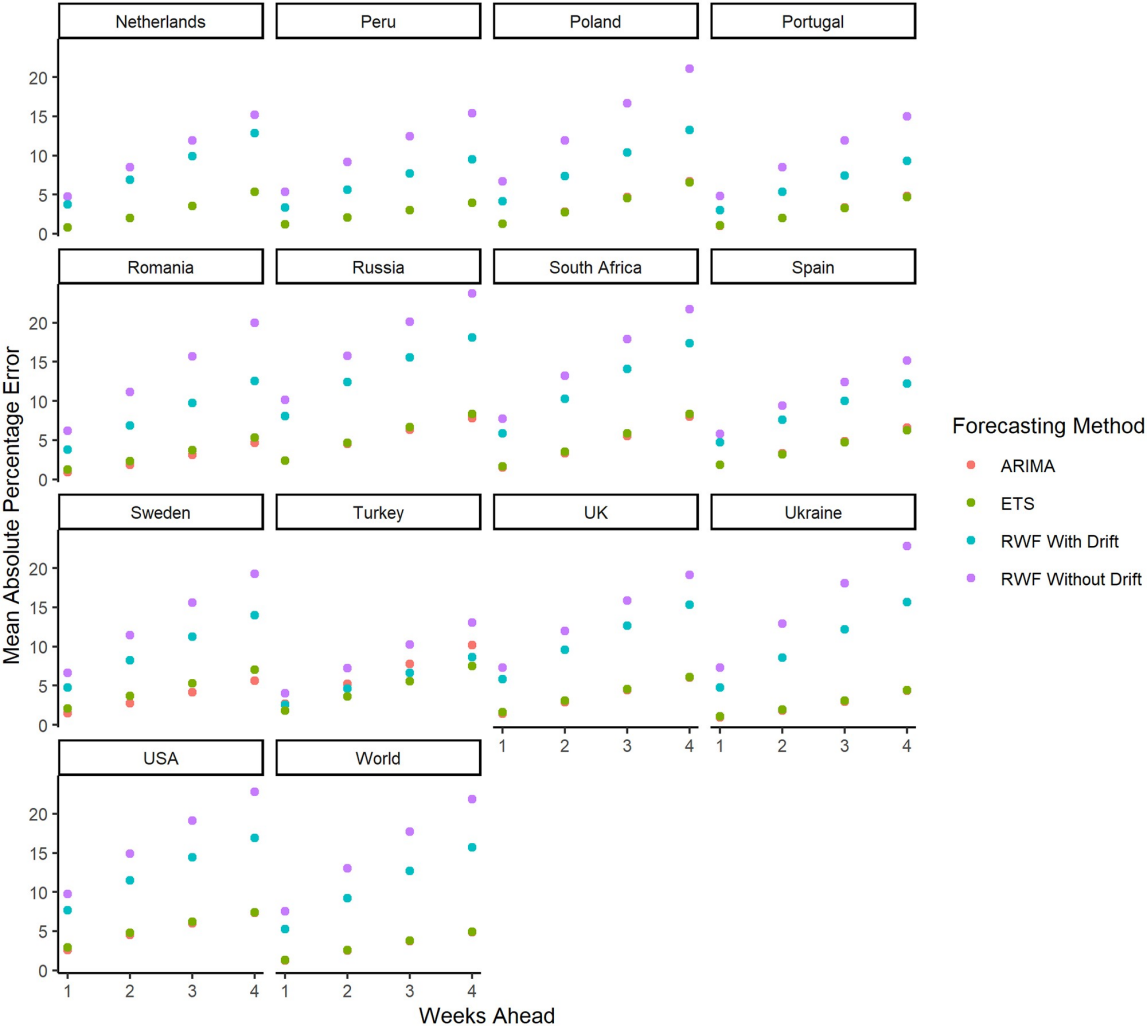
The *MAPE* for the forecasted values for 13 of the 29 countries with the highest cumulative COVID-19 cases and at the aggregated level for the entire world.

Figs 1 and 2 show the *MAPE* for the forecasted values of the 29 countries with the highest cumulative COVID-19 cases and at the aggregated level for the entire world. For all of the countries, the *MAPE* are provided in S1–S6 Figs. As expected, the *MAPE* increases as we increase the forecasting horizon from 1 week to 4 weeks ahead. This suggests that shorter-term forecasts are more accurate compared to longer-term forecasts. Overall, forecast evaluation shows that the *ARIMA* and *ETS* forecasts outperform *RWF* with and without drift.

Table 3 shows the summary of *MAPE* values of each forecasting horizon for the 187 countries and at the aggregated level for the entire world. In line with the observations of Figs 1 and 2, the *ARIMA* forecasts have low average *MAPE* values of 2.24%, 3.96%, 5.78%, and 7.65% for 1 week, 2 weeks, 3 weeks, and 4 weeks ahead, respectively. The *ETS* forecasts show similar average *MAPE* values as well. In comparison, *RWF* with drift has average *MAPE* values of 4.60% for 1 week, 7.89% for 2 weeks, 10.84% for 3 weeks, and 13.54% for 4 weeks ahead forecasts. Moreover, *RWF* without drift exhibits even higher values of *MAPE* than *RWF* with drift.

**Table 3:** Summary of the *MAPE* results for 187 countries and at the aggregated level of the world.

Weeks Ahead	Statistic	Forecasting Method			
		*ARIMA*	*ETS*	*RWF* With Drift	*RWF* Without Drift
1	Min.	0.11	0.10	1.46	0.08
	Max.	7.83	8.34	10.98	12.47
	Average	2.24	2.37	4.60	5.69
	St. Dev.	1.35	1.41	1.59	2.21
2	Min.	0.24	0.21	2.44	0.14
	Max.	11.81	13.44	18.5	21.01
	Average	3.96	4.18	7.89	9.80
	St. Dev.	2.07	2.24	2.61	3.72
3	Min.	0.37	0.34	3.23	0.16
	Max.	16.01	17.45	24.69	27.93
	Average	5.78	6.06	10.84	13.38
	St. Dev.	2.75	2.98	3.49	5.03
4	Min.	0.52	0.48	3.94	0.18
	Max.	20.01	20.55	29.79	33.62
	Average	7.65	7.97	13.54	16.58
	St. Dev.	3.42	3.69	4.29	6.20

**Fig 3:**
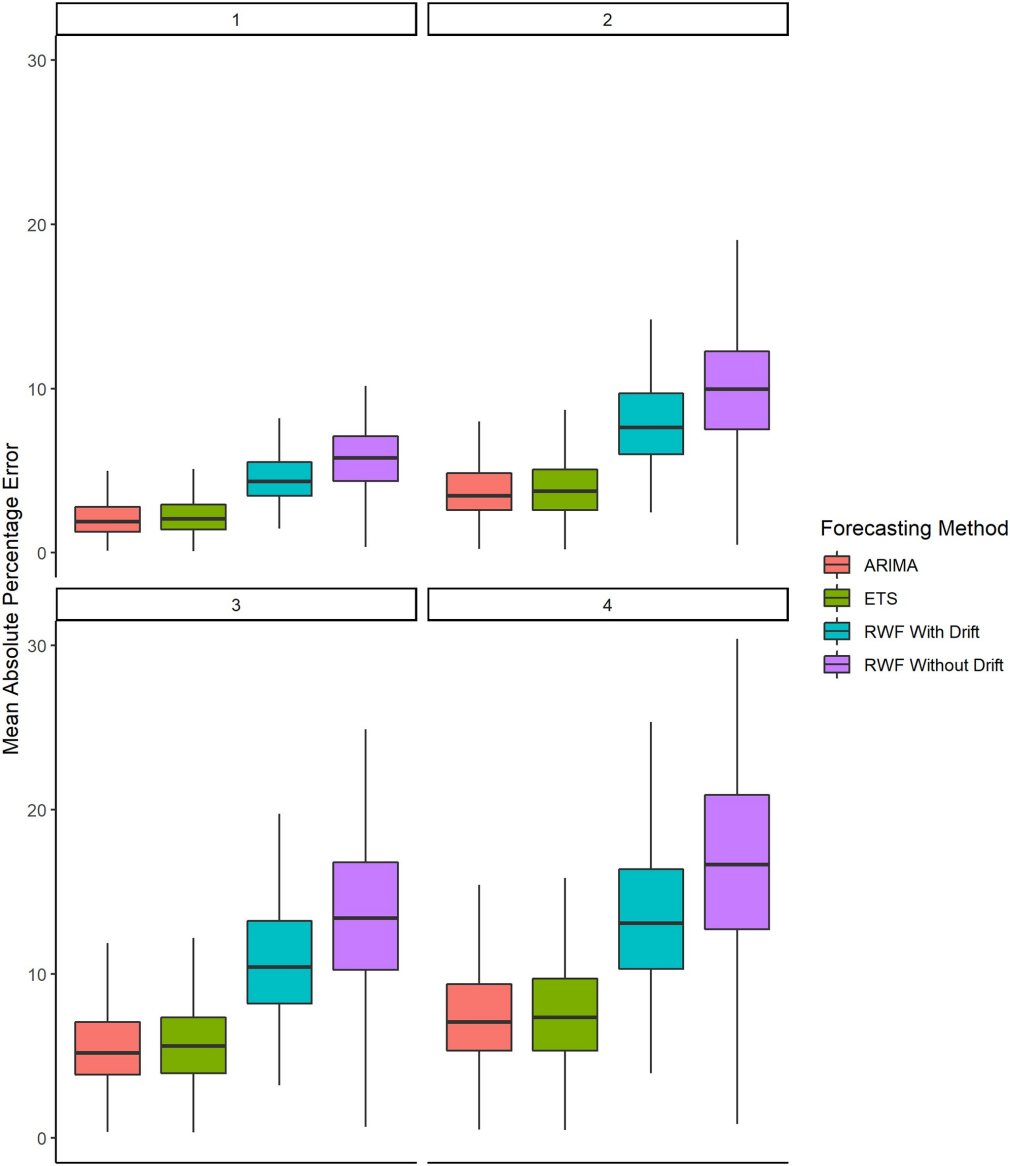
Boxplots of the *MAPE* results (without the outliers) for 187 countries and at the aggregated level of the world.

Fig 3 presents the *MAPE* results in the form of boxplots (without the outliers) for each of the forecasting methodology and horizon. A boxplot depicts the quartiles of the data. The box ranges from the first quartile (*Q*_1_) to the third quartile (*Q*_3_) and the black notch in the box represents the median of the data. Each boxplot also has a vertical line that encompasses the non-outliers of the data. The bottom and top limit of the vertical line are determined as *Q*_1_ − 1.5*IQR* and *Q*_3_ + 1.5*IQR*, respectively, where *IQR* = *Q*_3_ − *Q*_1_ is the interquartile range. Any observation beyond the vertical is referred to as an outlier because 99.3% of the observations lie within its limits.

Fig 3 shows that, among the non-outliers, the maximum *MAPE* values for 1 week, 2 weeks, 3 weeks, and 4 weeks ahead *ARIMA* forecasts are 4.97%, 8.00%, 11.89%, and 15.41%, respectively. Moreover, the median values for 1 week, 2 weeks, 3 weeks, and 4 weeks ahead forecasts are 1.88%, 3.47%, 5.20%, and 7.07%, respectively. The performance of *ARIMA* forecasts is marginally better than *ETS* forecasts and significantly better than *RWF* with and without drift.

**Fig 4:**
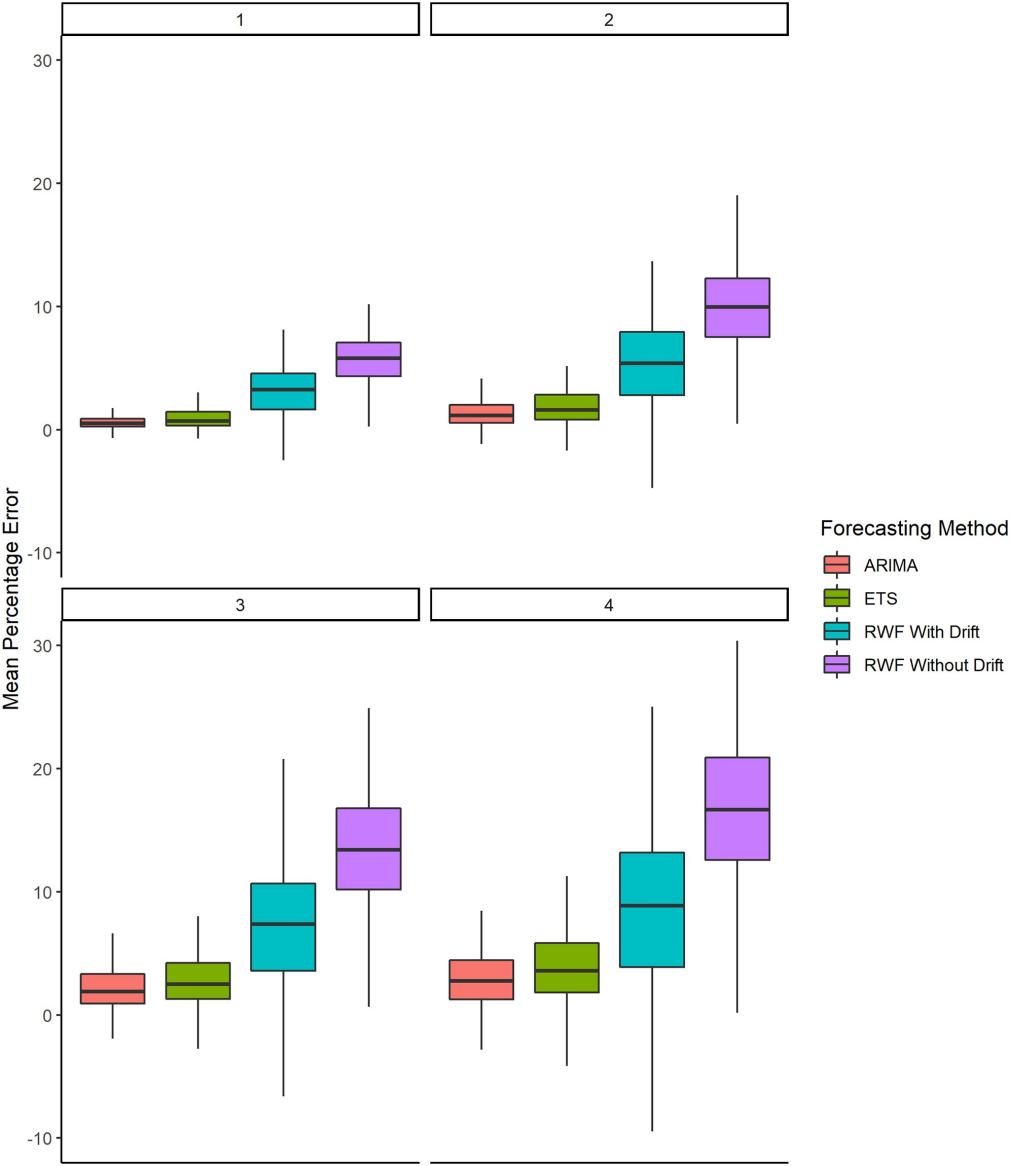
Boxplots of the *MPE* results (without the outliers) for 187 countries and at the aggregated level of the world.

Fig 4 shows the boxplots for the *MPE* of the forecasted values for each forecasting model. This figure shows that each forecasting model systematically over-predicts the cumulative COVID-19 cases. However, the over-predictions from the *RWF* with and without drift forecasts are much larger compared to the *ARIMA* and *ETS* forecasts.

We also used alternative measures for evaluating the forecasted values; Mean Absolute Error (*MAE*) and Mean Error (*ME*). Based on the results of these measures, presented in S1 File, the conclusions drawn from Figs 3 and 4 remain unchanged.

### Forecasted scenario

**Fig 5:**
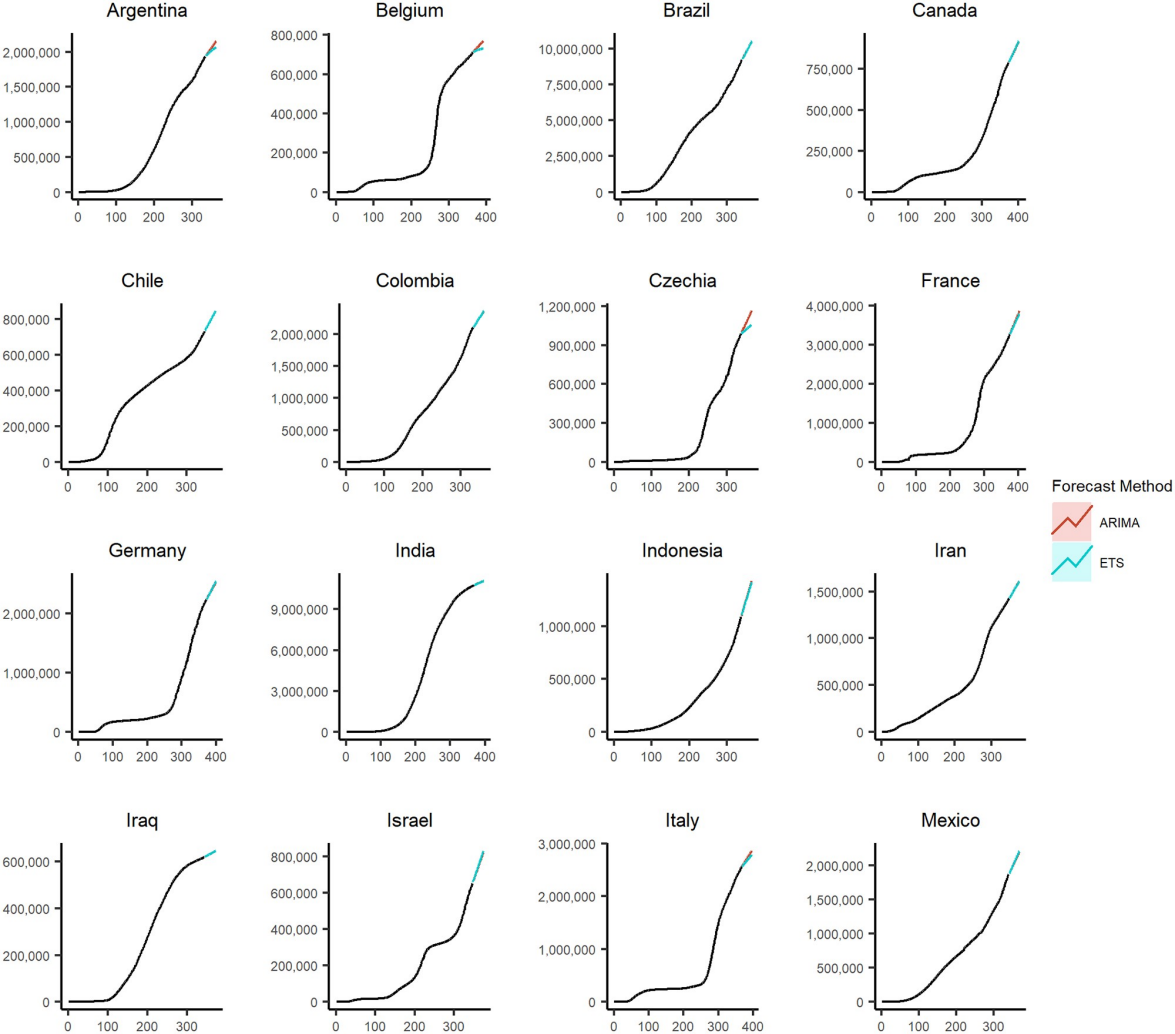
The 4 weeks ahead forecasts for 16 of the 29 countries with the highest cumulative COVID-19 cases.

**Fig 6:**
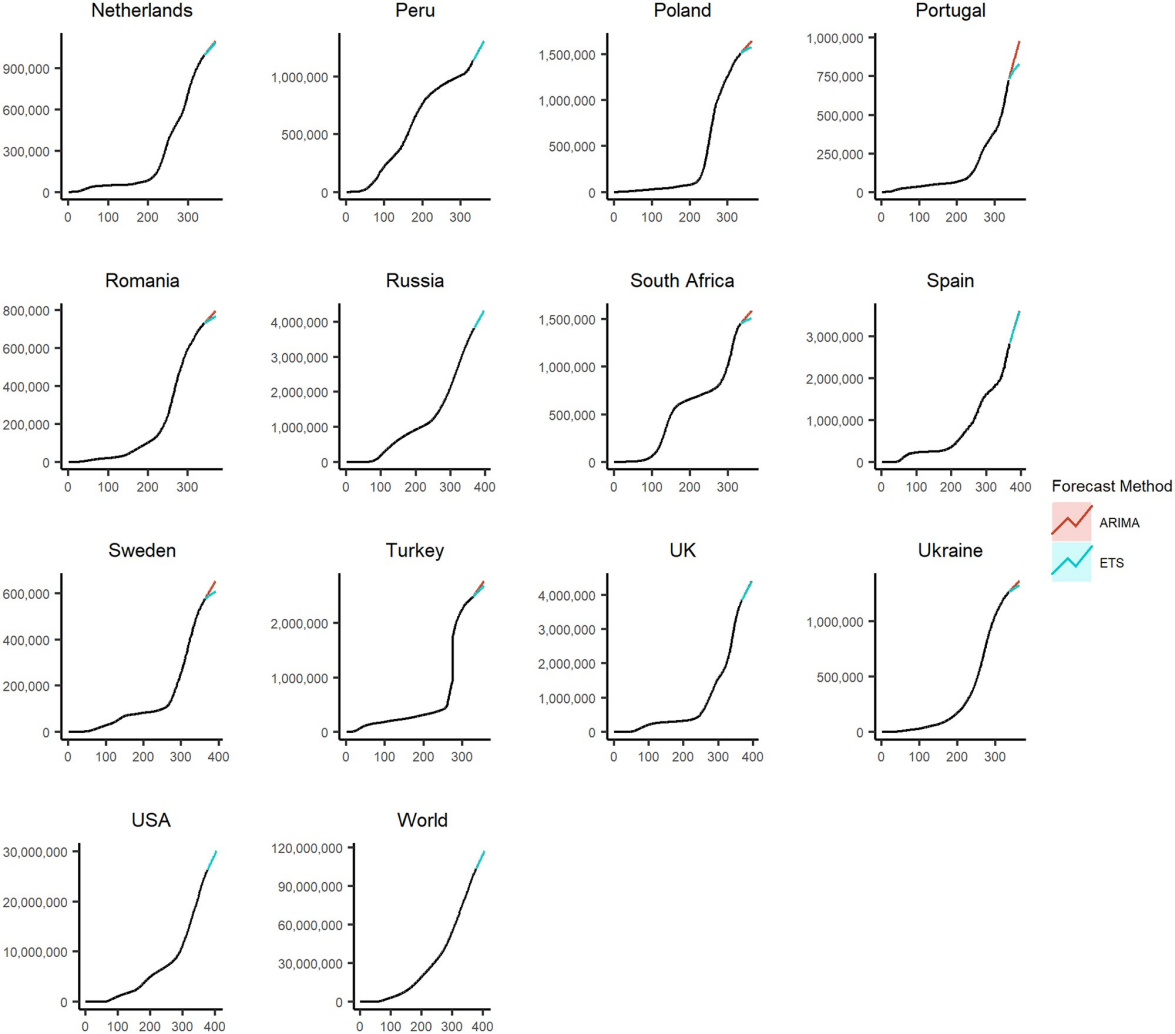
The 4 weeks ahead forecasts for 13 of the 29 countries with the highest cumulative COVID-19 cases and at the aggregated level for the entire world using the *ARIMA* and *ETS* methodologies.

Figs 5 and 6 show the forecasted values generated using the *ARIMA* and *ETS* forecasting methodologies for the 29 countries with the highest cumulative COVID-19 cases and at the aggregated level for the entire world. For all of the countries, the *ARIMA* and *ETS* forecasted values are provided in S7–S13 Figs.

We are only using the *ARIMA* and *ETS* forecasts as these outperform the *RWF* with and without drift as established in the previous subsection. These figures use the data from the entire sample period, and the values are forecasted for 4 weeks into the future. Figs 5 and 6 show that the *ARIMA* and *ETS* forecasts perform similarly in the depicted cases.

**Fig 7:**
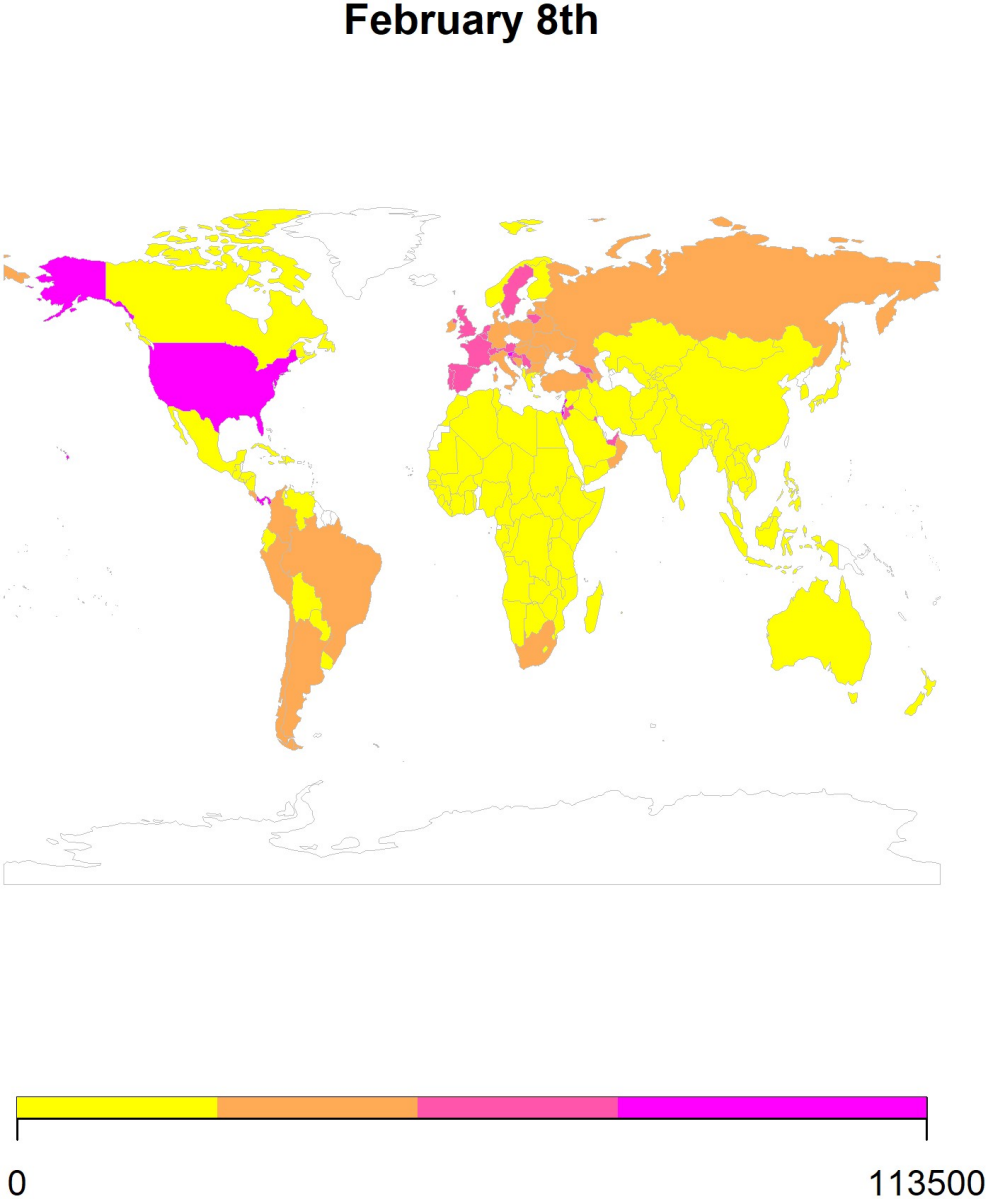
The heat map using the *ARIMA* forecasts for 8^th^ February 2021.

**Fig 8:**
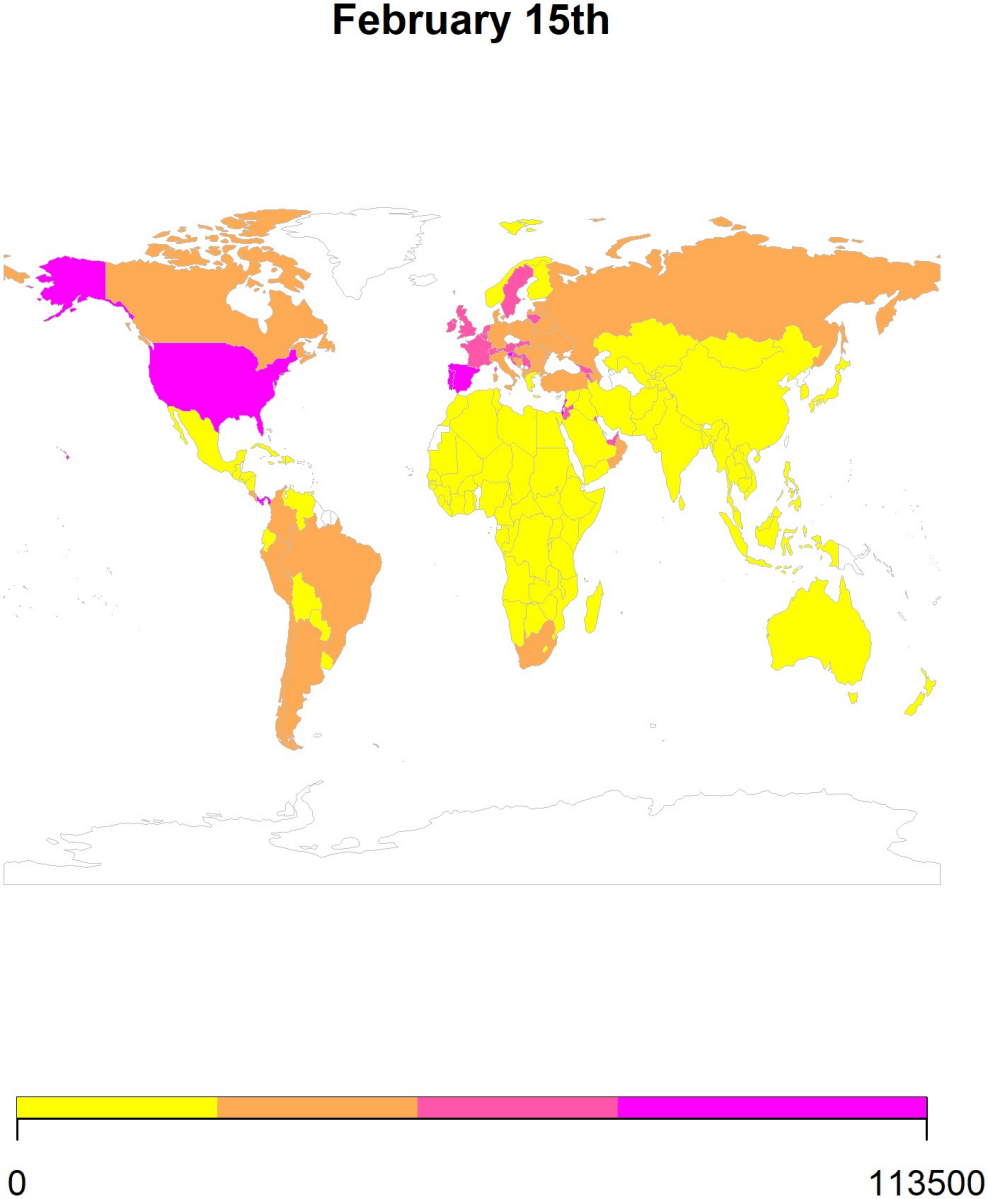
The heat map using the *ARIMA* forecasts for 15^th^ February 2021.

**Fig 9:**
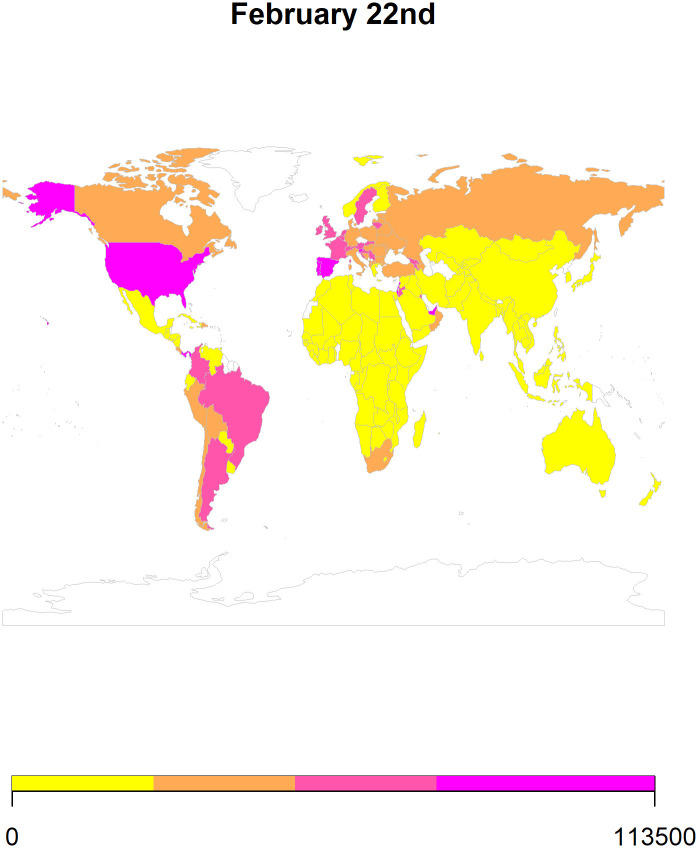
The heat map using the *ARIMA* forecasts for 22^nd^ February 2021.

**Fig 10:**
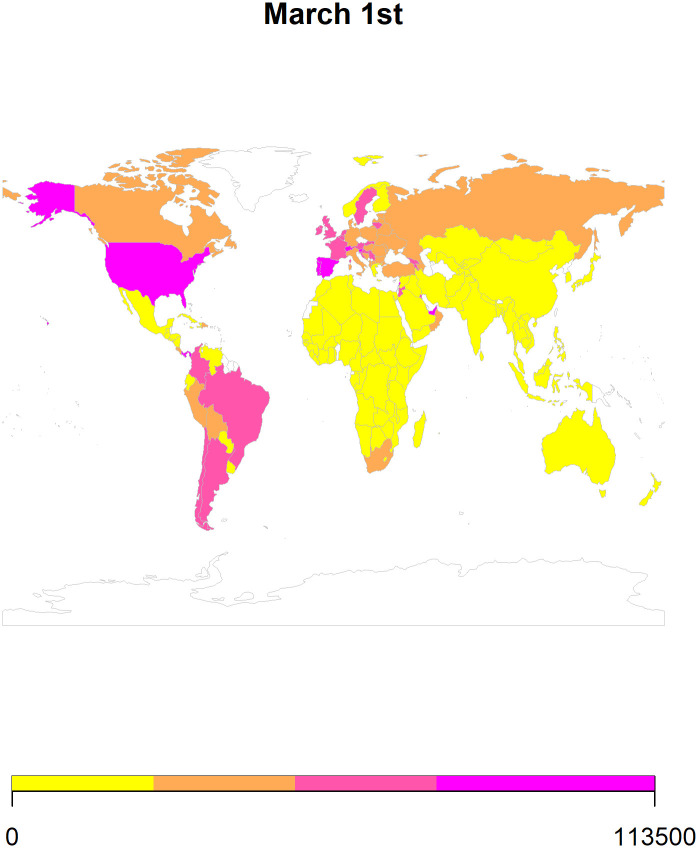
The heat map using the *ARIMA* forecasts for 1^st^ March 2021.

We also use *ARIMA* forecasts to generate heat maps for 8^th^ February (Fig 7), 15^th^ February (Fig 8), 22^nd^ February (Fig 9), and 1st March (Fig 10) in 2021. To generate the heat maps, we selected all those countries which had at least 73 (45+23) observations and have a population larger than 1 million. Moreover, we only use the *ARIMA* forecasts since its performance is comparable to the *ETS* forecasts. To ensure comparability of the forecasts, we divided the forecasted values of the cumulative COVID-19 cases by population in millions. Overall, the heat maps depict information for 145 countries as some of the countries were not matched with the countries listed in the rworldmap package used for generating these maps. The rworldmap package for R 4.0.3 uses the country borders from Natural Earth data v 1.4.0 which is in the public domain.

## Discussion

Our results show that the *ARIMA* and *ETS* methods perform well in forecasting cumulative COVID-19 cases. Additionally, using these forecasts, we generated heat maps to provide a pictorial representation of the countries at risk of having an increase in cases in the 4 weeks of February 2021.

Globally, uncertainty exists around the spread and transmissions of SARS-CoV2. For this purpose, many mathematical modeling and simulation-based techniques have been used, especially compartmental model techniques, to better understand the transmissions of COVID-19 cases. Among these, the most used is the Susceptible-Exposed-Infectious-Recovered (SEIR) model [21–24]. The SEIR model makes assumptions on the population belonging to the different compartments based on *R*_0_. However, for these assumptions to be reliable, large datasets are required and solely relying on *R*_0_ can be misleading as COVID-19 outbreaks may be possible even when *R*_0_ is lower than one [6, 21–24].

During a pandemic, not a lot of data is available to reliably run the aforementioned models. However, some of the models for infectious diseases were designed for determining long-term, instead of short-term, dynamics and projections [25]. In comparison, the data-driven methods considered in this paper are less data-hungry, perform well for short-term forecasts (based on evaluation of 4-week ahead forecasts), and do not require as much level of detail in the datasets. Other advantages of these data-driven techniques include simplicity of estimation that can be performed using the open-source statistical software R 4.0.3.

Different countries and regions have different health systems and capacities in place which determine their testing capabilities. The SEIR model can capture the propagation of the disease which means that it would be able to predict the true number of cases considering the susceptible and asymptomatic individuals. However, data for asymptomatic cases is largely unavailable for SARS-CoV2 due to limited testing capabilities and a large proportion of asymptomatic cases not being detected; making it challenging to verify the predictions from the compartmental models. On the other hand, data-driven techniques can provide information on the confirmed number of cases with high accuracy. For this study, we focused on the cumulative COVID-19 cases. However, these forecasting methods can be used for other indicators such as cumulative deaths, cumulative recovery, etc. The forecasts of the confirmed number of cases are sensitive to the number of tests performed, however, since the confirmed number of cases is an indicator of the anticipated burden on the healthcare system and professionals, the projections by the data-driven techniques might be insightful for the policymakers. This is important because the availability of health service resources during COVID-19 is an issue faced by many countries [26]. Even with lockdown measures enacted, the peak demand for healthcare services, during the COVID-19 pandemic, exceeded capacity irrespective of the capacity of the healthcare infrastructure and resources especially during the second wave [27, 28].

Globally, our forecasting results reveal that the number of cases will increase in most of the countries. Additionally, the forecasted scenarios for February 2021 indicate an increase in the cumulative cases of COVID-19 in Canada, Europe, and South America (Figs 7–10). The future of the global pandemic greatly depends on the vaccine rollout coupled with the implementation of mitigation and containment measures. Strict measures such as worldwide lockdowns, travel restrictions, school closures, non-essential business closures, social distancing, isolation of infected populations as well as heightened hygiene measures can potentially reduce the risk of spread [26]. However, the effectiveness of interventions is far from homogenous and depends on how well people comply, the presence of enforcement, how well testing/contact tracing/quarantine efforts that are run alongside the lockdown are performed, etc. Yet, hopes of curtailing the pandemic have proven elusive, with many countries forced by their economies to relax the quarantine measures which can potentially lead to an exponential increase in the number of cases. With effective vaccine rollout, close monitoring of COVID-19 cases should be considered before easing the mitigation and containment strategies.

Although the novel coronavirus pandemic is associated with many uncertainties, we believe that short-term forecasting and predictive modeling can be an effective tool in targeted vaccine rollout and intervention strategies. Model-based predictions can help policymakers to make the right decisions in a timely way [29].

## Conclusion

Results of the study indicate that the *ARIMA* and *ETS* models perform well in forecasting the short-term cumulative COVID-19 cases. We ran the model for 187 countries with varying health system resources and infrastructure, and at the aggregated level for the entire world. The results suggest that the *ARIMA* and *ETS* model can be used for SARS-CoV2 forecasting in different countries and regions with a high level of accuracy. Since these models rely on past observations of the cumulative COVID-19 cases, they can also be used for forecasting provincial, district, or state level cases and other COVID-19 indicators.

## Supporting information

S1 Data (CSV)

S1 File Alternative forecasting evaluation. (DOCX)

S1 Table Descriptive statistics of 187 countries and at the aggregated level of the entire world. (DOCX)

S2 Table Results of the unit root tests for 187 countries and at the aggregated level of the entire world. (DOCX)

S1 Fig The *MAPE* for the forecasted values of 187 countries and at the aggregated level for the entire world. (TIF)

S2 Fig The *MAPE* for the forecasted values of 187 countries and at the aggregated level for the entire world. (TIF)

S3 Fig The *MAPE* for the forecasted values of 187 countries and at the aggregated level for the entire world. (TIF)

S4 Fig The *MAPE* for the forecasted values of 187 countries and at the aggregated level for the entire world. (TIF)

S5 Fig The *MAPE* for the forecasted values of 187 countries and at the aggregated level for the entire world. (TIF)

S6 Fig The *MAPE* for the forecasted values of 187 countries and at the aggregated level for the entire world. (TIF)

S7 Fig The 4 weeks ahead forecasts for 187 countries and at the aggregated level for the entire world using the *ARIMA* and *ETS* methodologies. (TIF)

S8 Fig The 4 weeks ahead forecasts for 187 countries and at the aggregated level for the entire world using the *ARIMA* and *ETS* methodologies. (TIF)

S9 Fig The 4 weeks ahead forecasts for 187 countries and at the aggregated level for the entire world using the *ARIMA* and *ETS* methodologies. (TIF)

S10 Fig The 4 weeks ahead forecasts for 187 countries and at the aggregated level for the entire world using the *ARIMA* and *ETS* methodologies. (TIF)

S11 Fig The 4 weeks ahead forecasts for 187 countries and at the aggregated level for the entire world using the *ARIMA* and *ETS* methodologies. (TIF)

S12 Fig The 4 weeks ahead forecasts for 187 countries and at the aggregated level for the entire world using the *ARIMA* and *ETS* methodologies. (TIF)

S13 Fig The 4 weeks ahead forecasts for 187 countries and at the aggregated level for the entire world using the *ARIMA* and *ETS* methodologies. (TIF)
