# Supplementary material for: Evaluating data-driven methods for short-term forecasts of cumulative SARS-CoV2 cases
Source: PLoS One. 2021 May 21;16(5):e0252147. doi: 10.1371/journal.pone.0252147 (PMC8139504; doi:10.1371/journal.pone.0252147)

**S1 File. Alternative Forecasting Evaluation**

Instead of $MAPE$ and $MPE$, we also used the Mean Absolute Error ($MAE$) and Mean Error ($ME$) for evaluating the accuracy and systematic errors in the forecasted values. These are defined as follows: (Hyndman & Athanasopoulos, 2018)

$$\begin{aligned} MAE=\frac{1}{n}\sum_{i=1}^{n} \left| A_{i}-F_{i} \right|\#\left( 1 \right) \end{aligned}$$

$$\begin{aligned} ME=\frac{1}{n}\sum_{i=1}^{n} \left( A_{i}-F_{i} \right)\#\left( 2 \right) \end{aligned}$$

The boxplots of $MAE$ and $ME$ (without outliers) are presented in the figures below.


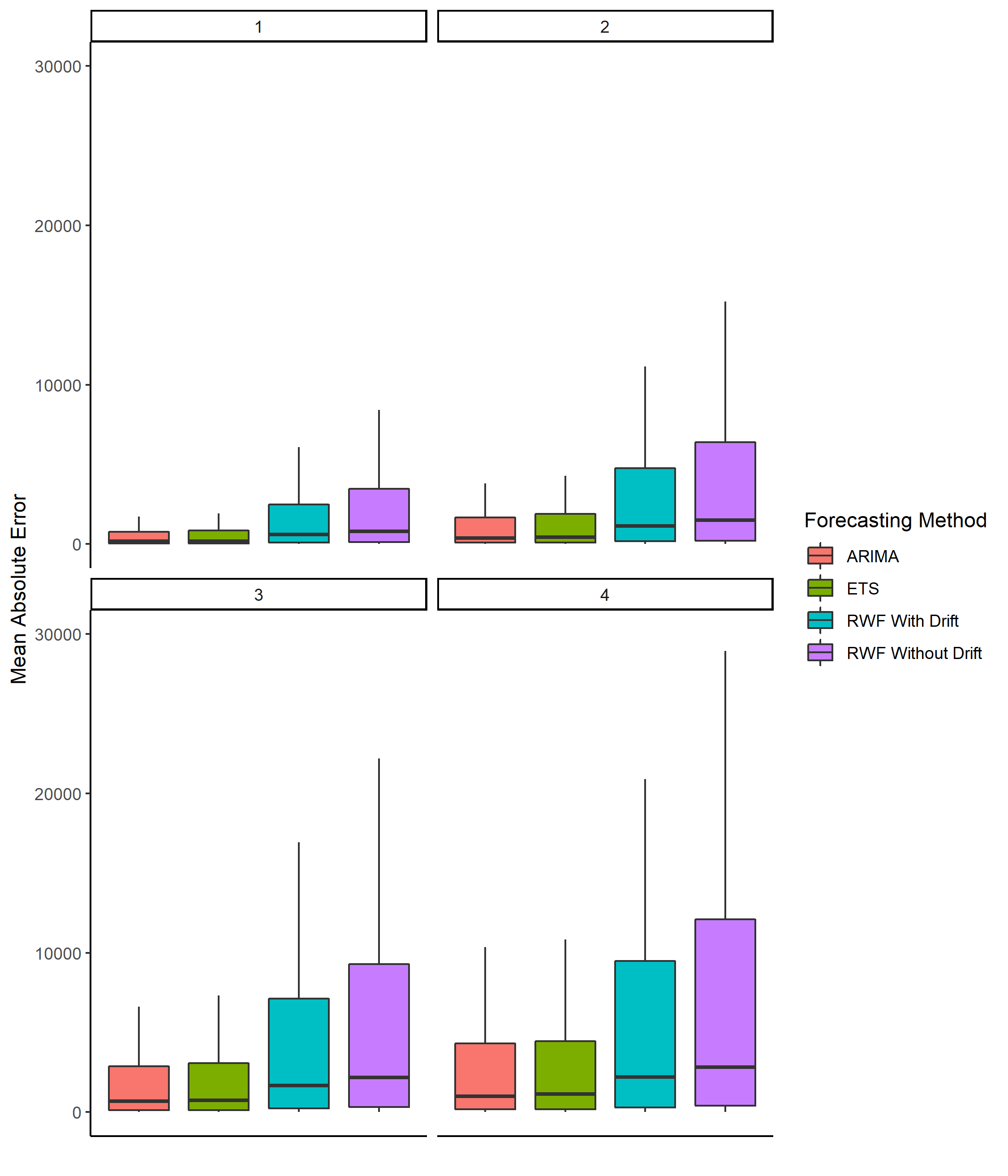


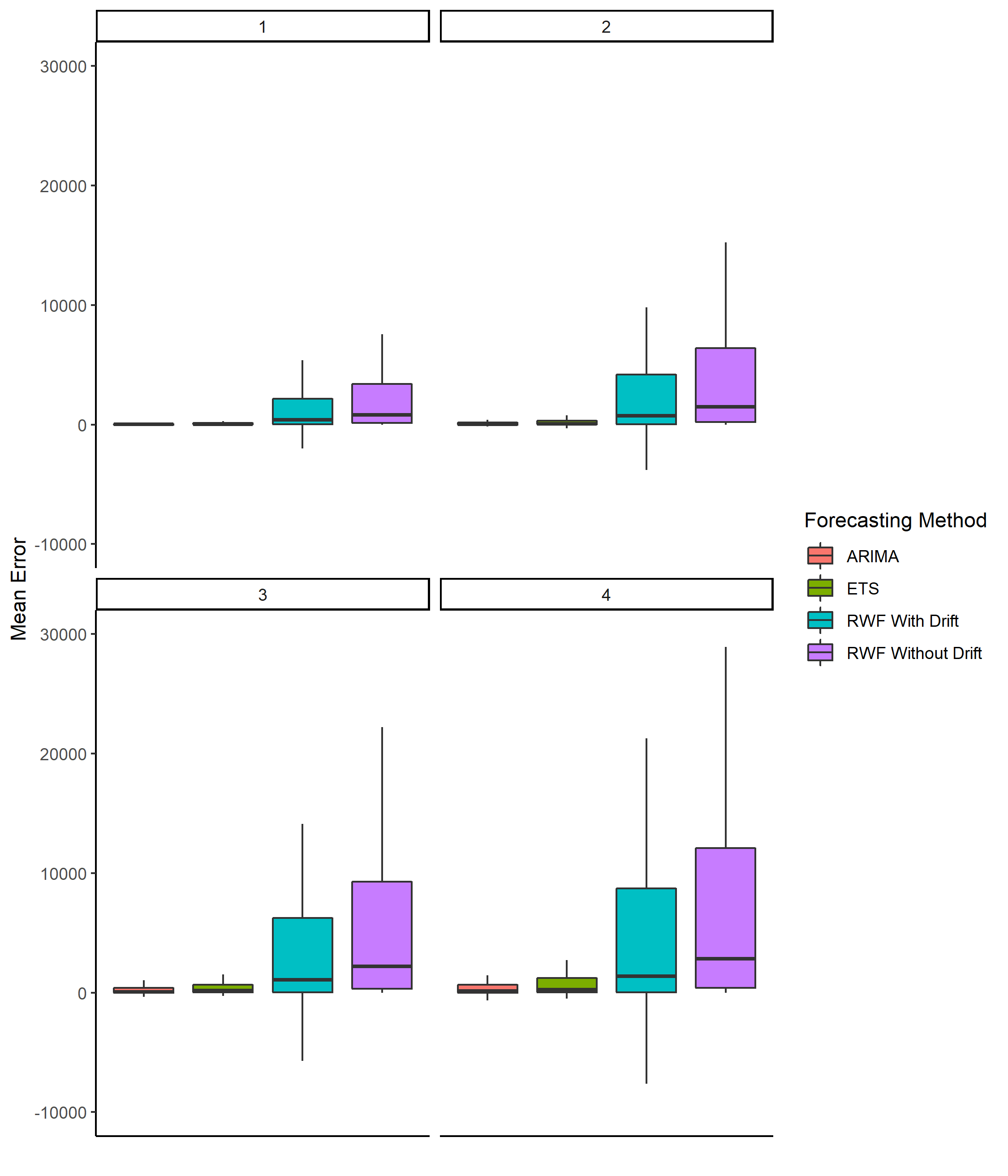

Supplement: S1 File — (DOCX) [file pone.0252147.s002.docx]
