# Supplementary material for: Evaluating data-driven methods for short-term forecasts of cumulative SARS-CoV2 cases
Source: PLoS One. 2021 May 21;16(5):e0252147. doi: 10.1371/journal.pone.0252147 (PMC8139504; doi:10.1371/journal.pone.0252147)
Supplement: S1 Table — (DOCX) [file pone.0252147.s003.docx]

**S1 Table.** Descriptive statistics of 187 countries and at the aggregated level of the entire world.

| **Country** | **Observations** | **Standard Deviation** | **Minimum** | **Maximum** |
| --- | --- | --- | --- | --- |
| Afghanistan | 344 | 18692.01 | 1 | 55059 |
| Albania | 330 | 22158.88 | 2 | 78992 |
| Algeria | 343 | 35233.47 | 1 | 107578 |
| Andorra | 337 | 3040.35 | 1 | 9972 |
| Angola | 319 | 7022.52 | 1 | 19829 |
| Antigua and Barbuda | 326 | 56.65 | 1 | 234 |
| Argentina | 336 | 654867.2 | 1 | 1933853 |
| Armenia | 338 | 57192.13 | 1 | 167088 |
| Australia | 373 | 11261.84 | 4 | 28823 |
| Austria | 343 | 135504.6 | 2 | 415522 |
| Azerbaijan | 338 | 74300.58 | 3 | 230296 |
| Bahamas | 323 | 3292.14 | 1 | 8174 |
| Bahrain | 344 | 35820.37 | 1 | 103582 |
| Bangladesh | 331 | 190476.4 | 3 | 535582 |
| Barbados | 322 | 301.68 | 2 | 1558 |
| Belarus | 340 | 65337.83 | 1 | 249295 |
| Belgium | 364 | 245428.4 | 1 | 711417 |
| Belize | 316 | 4026.78 | 1 | 11945 |
| Bhutan | 333 | 246.91 | 1 | 859 |
| Bolivia | 328 | 66812.17 | 2 | 218299 |
| Bosnia and Herzegovina | 334 | 41455.93 | 2 | 122199 |
| Botswana | 309 | 6166.26 | 3 | 22738 |
| Brazil | 342 | 2897244 | 1 | 9229322 |
| Brunei | 330 | 23.85 | 1 | 180 |
| Bulgaria | 331 | 75597.48 | 4 | 219580 |
| Burkina Faso | 329 | 2541.93 | 1 | 10768 |
| Burundi | 308 | 366.02 | 2 | 1635 |
| Cambodia | 372 | 127.78 | 1 | 466 |
| Cameroon | 333 | 9513.7 | 1 | 29617 |
| Canada | 373 | 206854.9 | 1 | 788186 |
| Cape Verde | 319 | 4658.55 | 1 | 14098 |
| Central African Republic | 324 | 2004.9 | 1 | 4989 |
| Chad | 320 | 781.81 | 1 | 3385 |
| Chile | 345 | 229784.1 | 2 | 730888 |
| China | 377 | 17652.86 | 548 | 100127 |
| Colombia | 333 | 644995.2 | 1 | 2104506 |
| Comoros | 278 | 507.45 | 1 | 2811 |
| Congo | 324 | 2577.09 | 1 | 7887 |
| Costa Rica | 333 | 66257.32 | 1 | 194569 |
| Cote d'Ivoire | 328 | 8887.74 | 1 | 28475 |
| Croatia | 343 | 77110.9 | 1 | 232520 |
| Cuba | 327 | 5265.54 | 3 | 27592 |
| Cyprus | 330 | 8759.74 | 2 | 30996 |
| Czechia | 338 | 295867.7 | 3 | 987329 |
| Democratic Republic of Congo | 328 | 6103.67 | 1 | 22842 |
| Denmark | 341 | 57395.46 | 1 | 199644 |
| Djibouti | 321 | 1963.09 | 1 | 5932 |
| Dominica | 317 | 32.96 | 1 | 117 |
| Dominican Republic | 338 | 64417.32 | 1 | 215086 |
| Ecuador | 338 | 76458.69 | 6 | 250986 |
| Egypt | 354 | 52121.18 | 1 | 166492 |
| El Salvador | 320 | 17436.46 | 1 | 55195 |
| Equatorial Guinea | 324 | 2086.34 | 1 | 5534 |
| Eritrea | 318 | 507.32 | 1 | 2135 |
| Estonia | 341 | 10995.53 | 1 | 44575 |
| Eswatini | 325 | 3937.72 | 1 | 15804 |
| Ethiopia | 326 | 50119.11 | 1 | 138384 |
| Finland | 370 | 12182.69 | 1 | 45482 |
| France | 375 | 1017765 | 2 | 3260308 |
| Gabon | 325 | 3645.22 | 1 | 10952 |
| Gambia | 322 | 1756.59 | 1 | 4139 |
| Georgia | 342 | 85473.74 | 1 | 258351 |
| Germany | 372 | 613919.7 | 1 | 2232327 |
| Ghana | 325 | 21255.85 | 3 | 67010 |
| Greece | 342 | 52396.5 | 1 | 157495 |
| Grenada | 317 | 38.39 | 1 | 148 |
| Guatemala | 325 | 53593.87 | 1 | 159632 |
| Guinea | 326 | 4814.15 | 1 | 14555 |
| Guinea-Bissau | 314 | 817.75 | 2 | 2634 |
| Guyana | 327 | 2540.21 | 1 | 7654 |
| Haiti | 319 | 3779.58 | 2 | 11533 |
| Honduras | 328 | 47581.09 | 2 | 147843 |
| Hungary | 335 | 122834 | 2 | 368710 |
| Iceland | 340 | 1821.15 | 1 | 6009 |
| India | 369 | 4124427 | 1 | 10766245 |
| Indonesia | 337 | 292201.6 | 2 | 1089308 |
| Iran | 349 | 421107.1 | 2 | 1424596 |
| Iraq | 344 | 237033.7 | 1 | 620620 |
| Ireland | 339 | 44360.02 | 1 | 197553 |
| Israel | 347 | 179182.7 | 1 | 652246 |
| Italy | 368 | 755731.6 | 2 | 2560957 |
| Jamaica | 328 | 5159.39 | 1 | 15778 |
| Japan | 377 | 94481.24 | 2 | 391956 |
| Jordan | 336 | 114188 | 1 | 328062 |
| Kazakhstan | 326 | 76994.08 | 4 | 237029 |
| Kenya | 326 | 35802.48 | 1 | 100856 |
| Kosovo | 325 | 18873.7 | 2 | 60218 |
| Kuwait | 344 | 56602.48 | 1 | 165843 |
| Kyrgyzstan | 321 | 30790.93 | 3 | 84588 |
| Laos | 315 | 9.34 | 2 | 44 |
| Latvia | 337 | 16918.51 | 1 | 66652 |
| Lebanon | 347 | 79194.6 | 1 | 303072 |
| Lesotho | 265 | 1991.31 | 1 | 8900 |
| Liberia | 323 | 626.95 | 1 | 1944 |
| Libya | 315 | 40407.65 | 1 | 119402 |
| Liechtenstein | 335 | 780.81 | 1 | 2497 |
| Lithuania | 339 | 53803.7 | 1 | 182898 |
| Luxembourg | 339 | 16236.59 | 1 | 50733 |
| Madagascar | 319 | 7575.91 | 3 | 19065 |
| Malawi | 306 | 4413.82 | 3 | 24365 |
| Malaysia | 374 | 47608.47 | 3 | 219173 |
| Maldives | 331 | 5425.37 | 4 | 16056 |
| Mali | 314 | 2216.04 | 2 | 8100 |
| Malta | 332 | 5114.79 | 3 | 18027 |
| Marshall Islands | 97 | 1.08 | 1 | 4 |
| Mauritania | 325 | 4878.68 | 1 | 16662 |
| Mauritius | 321 | 109.59 | 3 | 582 |
| Mexico | 340 | 536228.6 | 1 | 1869708 |
| Micronesia (country) | 12 | 0 | 1 | 1 |
| Moldova | 331 | 51270.3 | 1 | 160086 |
| Monaco | 339 | 355.57 | 1 | 1499 |
| Mongolia | 329 | 449.41 | 1 | 1832 |
| Montenegro | 322 | 18945.6 | 2 | 61972 |
| Morocco | 337 | 166255.4 | 1 | 471438 |
| Mozambique | 317 | 9045.89 | 1 | 39460 |
| Myanmar | 312 | 49328.25 | 8 | 140354 |
| Namibia | 325 | 9537.27 | 2 | 34168 |
| Nepal | 374 | 100989.4 | 1 | 271118 |
| Netherlands | 341 | 298779.7 | 1 | 995300 |
| New Zealand | 340 | 569.49 | 1 | 2307 |
| Nicaragua | 320 | 2315.11 | 1 | 6253 |
| Niger | 319 | 1037.55 | 1 | 4537 |
| Nigeria | 340 | 34247.36 | 1 | 131918 |
| North Macedonia | 342 | 30104.3 | 1 | 92753 |
| Norway | 342 | 17054.37 | 1 | 63262 |
| Oman | 344 | 50701.79 | 2 | 134524 |
| Pakistan | 343 | 170293.7 | 2 | 547648 |
| Palestine | 334 | 50157.93 | 4 | 159443 |
| Panama | 329 | 89974.94 | 1 | 321103 |
| Paraguay | 331 | 42623.23 | 1 | 133781 |
| Peru | 333 | 390101.4 | 1 | 1138239 |
| Philippines | 369 | 190087.3 | 1 | 527272 |
| Poland | 335 | 503521.2 | 1 | 1515889 |
| Portugal | 337 | 172793.4 | 2 | 726321 |
| Qatar | 339 | 52842.89 | 1 | 151720 |
| Romania | 342 | 235456 | 1 | 730056 |
| Russia | 368 | 1092717 | 2 | 3825739 |
| Rwanda | 325 | 3529.23 | 1 | 15459 |
| Saint Kitts and Nevis | 314 | 6.94 | 2 | 38 |
| Saint Lucia | 325 | 220.29 | 1 | 1411 |
| Saint Vincent and the Grenadines | 325 | 158.95 | 1 | 901 |
| Samoa | 76 | 0.33 | 1 | 2 |
| San Marino | 341 | 750.26 | 1 | 3025 |
| Sao Tome and Principe | 302 | 334.54 | 4 | 1259 |
| Saudi Arabia | 337 | 141471.9 | 1 | 368329 |
| Senegal | 337 | 7350.55 | 1 | 26927 |
| Serbia | 333 | 121900.8 | 1 | 397002 |
| Seychelles | 325 | 224.44 | 2 | 1223 |
| Sierra Leone | 308 | 905.68 | 1 | 3634 |
| Singapore | 376 | 23672.34 | 1 | 59565 |
| Slovakia | 333 | 72449.16 | 1 | 250357 |
| Slovenia | 334 | 49068.29 | 2 | 166836 |
| Solomon Islands | 113 | 4.89 | 2 | 17 |
| Somalia | 323 | 1521.49 | 1 | 4784 |
| South Africa | 334 | 418621.3 | 1 | 1456309 |
| South Korea | 377 | 19486.44 | 1 | 78844 |
| South Sudan | 303 | 1140.86 | 1 | 3929 |
| Spain | 367 | 743438.9 | 1 | 2822805 |
| Sri Lanka | 372 | 16559.27 | 1 | 64983 |
| Sudan | 325 | 8339.34 | 1 | 29449 |
| Suriname | 325 | 2667.6 | 1 | 8449 |
| Sweden | 364 | 157663.7 | 1 | 576606 |
| Switzerland | 343 | 165093 | 1 | 525095 |
| Syria | 317 | 4353.29 | 1 | 14096 |
| Taiwan | 377 | 231.24 | 1 | 912 |
| Tajikistan | 278 | 3693.2 | 15 | 13308 |
| Tanzania | 323 | 150.55 | 1 | 509 |
| Thailand | 377 | 3207.12 | 4 | 20454 |
| Timor | 317 | 12.37 | 1 | 70 |
| Togo | 333 | 1376.24 | 1 | 5092 |
| Trinidad and Tobago | 325 | 2991.5 | 2 | 7566 |
| Tunisia | 335 | 58918.87 | 1 | 210045 |
| Turkey | 328 | 755429.5 | 1 | 2485182 |
| UAE | 370 | 78873.7 | 4 | 306339 |
| UK | 368 | 983256 | 2 | 3846851 |
| USA | 377 | 7199436 | 1 | 26321120 |
| Uganda | 318 | 12912.26 | 1 | 39606 |
| Ukraine | 336 | 402486.9 | 1 | 1263833 |
| Uruguay | 326 | 9466.96 | 4 | 42128 |
| Uzbekistan | 324 | 30544.64 | 1 | 78755 |
| Vanuatu | 84 | 0 | 1 | 1 |
| Vatican | 333 | 8.29 | 1 | 27 |
| Venezuela | 325 | 46292.54 | 2 | 127346 |
| Vietnam | 376 | 537.29 | 2 | 1850 |
| World | 377 | 30371381 | 557 | 103422636 |
| Yemen | 298 | 773.86 | 1 | 2122 |
| Zambia | 321 | 11491.57 | 2 | 55042 |
