# Supplementary material for: Evaluating data-driven methods for short-term forecasts of cumulative SARS-CoV2 cases
Source: PLoS One. 2021 May 21;16(5):e0252147. doi: 10.1371/journal.pone.0252147 (PMC8139504; doi:10.1371/journal.pone.0252147)
Supplement: S2 Table — (DOCX) [file pone.0252147.s004.docx]

**S2 Table.** Results of the unit root tests for 187 countries and at the aggregated level of the entire world.

| **Country** | $\boldsymbol{ADF}$^a^ | $\boldsymbol{PP}$^b^ |
| --- | --- | --- |
| Afghanistan | 0.38 | 0.75 |
| Albania | 1.00 | 1.00 |
| Algeria | 1.00 | 1.00 |
| Andorra | 1.00 | 1.00 |
| Angola | 1.00 | 1.00 |
| Antigua and Barbuda | 1.00 | 1.00 |
| Argentina | 1.00 | 1.00 |
| Armenia | 1.00 | 1.00 |
| Australia | 0.36 | 0.75 |
| Austria | 1.00 | 1.00 |
| Azerbaijan | 1.00 | 1.00 |
| Bahamas | 1.00 | 1.00 |
| Bahrain | 1.00 | 1.00 |
| Bangladesh | 1.00 | 0.98 |
| Barbados | 1.00 | 1.00 |
| Belarus | 1.00 | 1.00 |
| Belgium | 1.00 | 1.00 |
| Belize | 1.00 | 1.00 |
| Bhutan | 1.00 | 1.00 |
| Bolivia | 1.00 | 1.00 |
| Bosnia and Herzegovina | 1.00 | 1.00 |
| Botswana | 1.00 | 1.00 |
| Brazil | 1.00 | 1.00 |
| Brunei | 0.00 | 0.00 |
| Bulgaria | 1.00 | 1.00 |
| Burkina Faso | 1.00 | 1.00 |
| Burundi | 1.00 | 1.00 |
| Cambodia | 1.00 | 0.99 |
| Cameroon | 0.85 | 0.85 |
| Canada | 1.00 | 1.00 |
| Cape Verde | 1.00 | 1.00 |
| Central African Republic | 0.00 | 0.18 |
| Chad | 1.00 | 1.00 |
| Chile | 1.00 | 1.00 |
| China | 0.00 | 0.00 |
| Colombia | 1.00 | 1.00 |
| Comoros | 1.00 | 1.00 |
| Congo | 0.99 | 0.99 |
| Costa Rica | 1.00 | 1.00 |
| Cote d'Ivoire | 0.99 | 0.97 |
| Croatia | 1.00 | 1.00 |
| Cuba | 1.00 | 1.00 |
| Cyprus | 1.00 | 1.00 |
| Czechia | 1.00 | 1.00 |
| Democratic Republic of Congo | 1.00 | 1.00 |
| Denmark | 1.00 | 1.00 |
| Djibouti | 0.00 | 0.01 |
| Dominica | 1.00 | 1.00 |
| Dominican Republic | 1.00 | 1.00 |
| Ecuador | 1.00 | 1.00 |
| Egypt | 1.00 | 1.00 |
| El Salvador | 1.00 | 1.00 |
| Equatorial Guinea | 0.61 | 0.60 |
| Eritrea | 1.00 | 1.00 |
| Estonia | 1.00 | 1.00 |
| Eswatini | 1.00 | 1.00 |
| Ethiopia | 1.00 | 1.00 |
| Finland | 1.00 | 1.00 |
| France | 1.00 | 1.00 |
| Gabon | 0.20 | 0.42 |
| Gambia | 0.97 | 0.95 |
| Georgia | 1.00 | 1.00 |
| Germany | 1.00 | 1.00 |
| Ghana | 0.99 | 0.97 |
| Greece | 1.00 | 1.00 |
| Grenada | 1.00 | 1.00 |
| Guatemala | 1.00 | 1.00 |
| Guinea | 0.06 | 0.22 |
| Guinea-Bissau | 0.00 | 0.00 |
| Guyana | 1.00 | 1.00 |
| Haiti | 0.22 | 0.64 |
| Honduras | 1.00 | 1.00 |
| Hungary | 1.00 | 1.00 |
| Iceland | 0.98 | 0.95 |
| India | 1.00 | 1.00 |
| Indonesia | 1.00 | 1.00 |
| Iran | 1.00 | 1.00 |
| Iraq | 1.00 | 1.00 |
| Ireland | 1.00 | 1.00 |
| Israel | 1.00 | 1.00 |
| Italy | 1.00 | 1.00 |
| Jamaica | 1.00 | 1.00 |
| Japan | 1.00 | 1.00 |
| Jordan | 1.00 | 1.00 |
| Kazakhstan | 1.00 | 1.00 |
| Kenya | 1.00 | 1.00 |
| Kosovo | 1.00 | 1.00 |
| Kuwait | 1.00 | 1.00 |
| Kyrgyzstan | 0.99 | 0.99 |
| Laos | 0.60 | 0.59 |
| Latvia | 1.00 | 1.00 |
| Lebanon | 1.00 | 1.00 |
| Lesotho | 1.00 | 1.00 |
| Liberia | 0.66 | 0.74 |
| Libya | 1.00 | 1.00 |
| Liechtenstein | 1.00 | 1.00 |
| Lithuania | 1.00 | 1.00 |
| Luxembourg | 1.00 | 1.00 |
| Madagascar | 0.64 | 0.82 |
| Malawi | 1.00 | 1.00 |
| Malaysia | 1.00 | 1.00 |
| Maldives | 1.00 | 1.00 |
| Mali | 1.00 | 1.00 |
| Malta | 1.00 | 1.00 |
| Marshall Islands | 0.41 | 0.39 |
| Mauritania | 1.00 | 1.00 |
| Mauritius | 0.00 | 0.00 |
| Mexico | 1.00 | 1.00 |
| Micronesia (country) | 1.00 | 1.00 |
| Moldova | 1.00 | 1.00 |
| Monaco | 1.00 | 1.00 |
| Mongolia | 1.00 | 1.00 |
| Montenegro | 1.00 | 1.00 |
| Morocco | 1.00 | 1.00 |
| Mozambique | 1.00 | 1.00 |
| Myanmar | 1.00 | 1.00 |
| Namibia | 1.00 | 1.00 |
| Nepal | 1.00 | 1.00 |
| Netherlands | 1.00 | 1.00 |
| New Zealand | 0.00 | 0.01 |
| Nicaragua | 0.64 | 0.51 |
| Niger | 1.00 | 1.00 |
| Nigeria | 1.00 | 1.00 |
| North Macedonia | 1.00 | 1.00 |
| Norway | 1.00 | 1.00 |
| Oman | 0.94 | 0.93 |
| Pakistan | 1.00 | 1.00 |
| Palestine | 1.00 | 1.00 |
| Panama | 1.00 | 1.00 |
| Paraguay | 1.00 | 1.00 |
| Peru | 1.00 | 0.99 |
| Philippines | 1.00 | 1.00 |
| Poland | 1.00 | 1.00 |
| Portugal | 1.00 | 1.00 |
| Qatar | 0.00 | 0.11 |
| Romania | 1.00 | 1.00 |
| Russia | 1.00 | 1.00 |
| Rwanda | 1.00 | 1.00 |
| Saint Kitts and Nevis | 0.94 | 0.94 |
| Saint Lucia | 1.00 | 1.00 |
| Saint Vincent and the Grenadines | 1.00 | 1.00 |
| Samoa | 0.05 | 0.05 |
| San Marino | 1.00 | 1.00 |
| Sao Tome and Principe | 0.18 | 0.24 |
| Saudi Arabia | 0.00 | 0.14 |
| Senegal | 1.00 | 1.00 |
| Serbia | 1.00 | 1.00 |
| Seychelles | 1.00 | 1.00 |
| Sierra Leone | 0.99 | 0.97 |
| Singapore | 0.00 | 0.19 |
| Slovakia | 1.00 | 1.00 |
| Slovenia | 1.00 | 1.00 |
| Solomon Islands | 0.05 | 0.02 |
| Somalia | 0.00 | 0.03 |
| South Africa | 1.00 | 1.00 |
| South Korea | 1.00 | 1.00 |
| South Sudan | 0.36 | 0.48 |
| Spain | 1.00 | 1.00 |
| Sri Lanka | 1.00 | 1.00 |
| Sudan | 1.00 | 1.00 |
| Suriname | 1.00 | 1.00 |
| Sweden | 1.00 | 1.00 |
| Switzerland | 1.00 | 1.00 |
| Syria | 1.00 | 1.00 |
| Taiwan | 0.93 | 0.91 |
| Tajikistan | 0.00 | 0.00 |
| Tanzania | 0.00 | 0.01 |
| Thailand | 1.00 | 1.00 |
| Timor | 1.00 | 0.99 |
| Togo | 1.00 | 1.00 |
| Trinidad and Tobago | 1.00 | 1.00 |
| Tunisia | 1.00 | 1.00 |
| Turkey | 1.00 | 1.00 |
| UAE | 1.00 | 1.00 |
| UK | 1.00 | 1.00 |
| USA | 1.00 | 1.00 |
| Uganda | 1.00 | 1.00 |
| Ukraine | 1.00 | 1.00 |
| Uruguay | 1.00 | 1.00 |
| Uzbekistan | 1.00 | 0.98 |
| Vanuatu | 1.00 | 1.00 |
| Vatican | 0.67 | 0.65 |
| Venezuela | 1.00 | 1.00 |
| Vietnam | 1.00 | 1.00 |
| World | 1.00 | 1.00 |
| Yemen | 0.00 | 0.01 |
| Zambia | 1.00 | 1.00 |

^a^ The p-values of the Augmented Dickey-Fuller unit root test.

^b^ The p-values of the Phillips-Perron unit root test.
