# Supplementary figures and images for: Evaluating data-driven methods for short-term forecasts of cumulative SARS-CoV2 cases
Source: PLoS One. 2021 May 21;16(5):e0252147. doi: 10.1371/journal.pone.0252147 (PMC8139504; doi:10.1371/journal.pone.0252147)

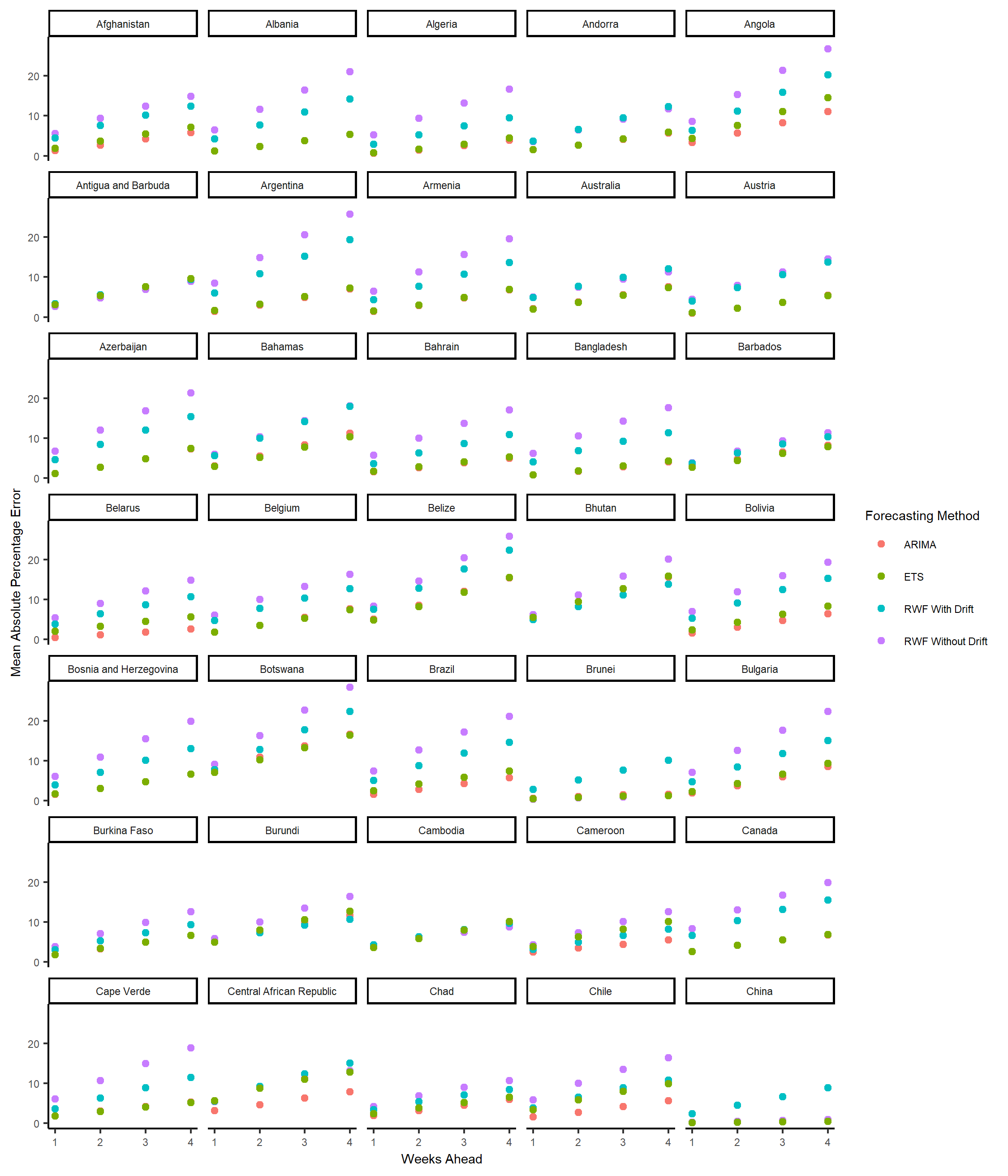

Supplement: S1 Fig — (TIF) [file pone.0252147.s005.tif]

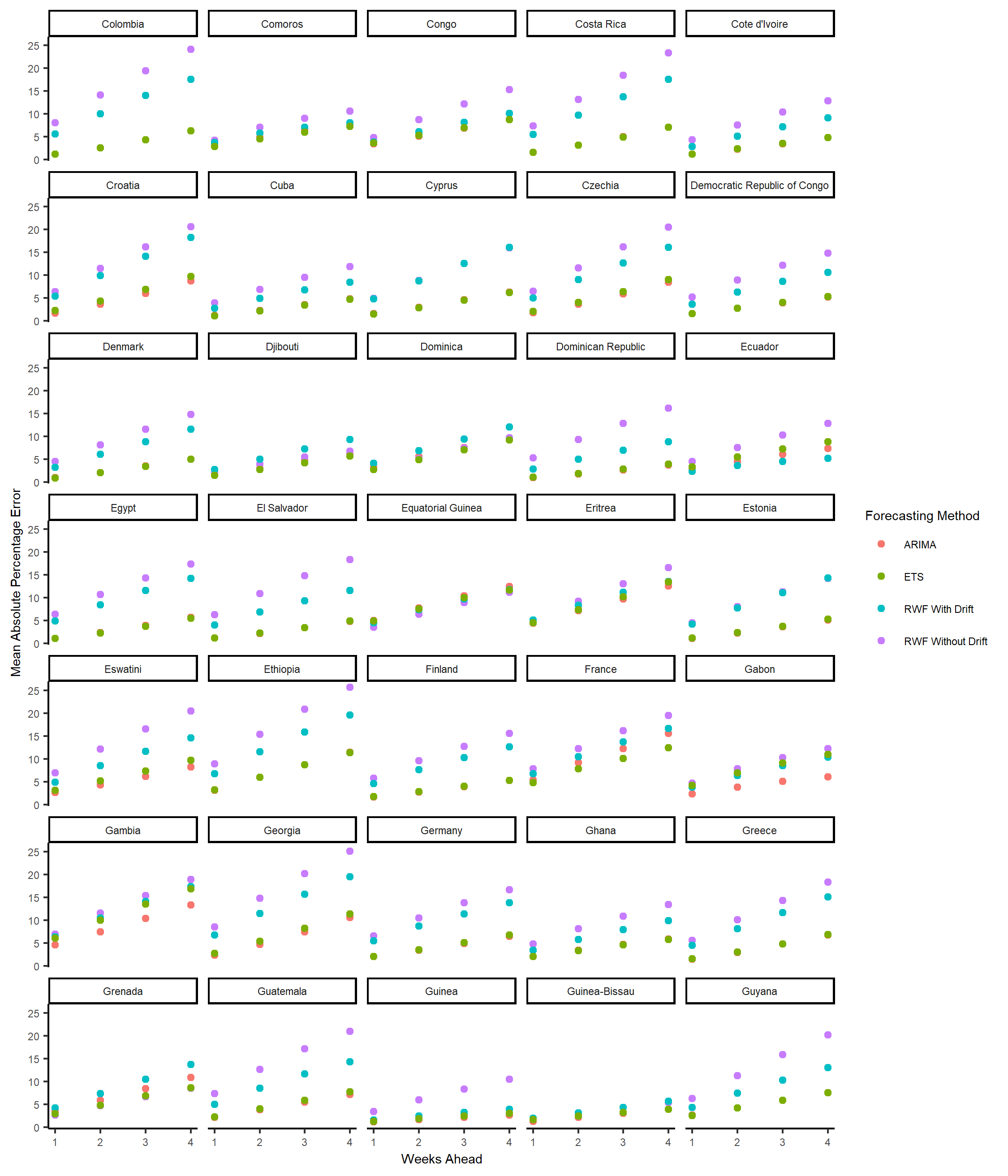

Supplement: S2 Fig — (TIF) [file pone.0252147.s006.tif]

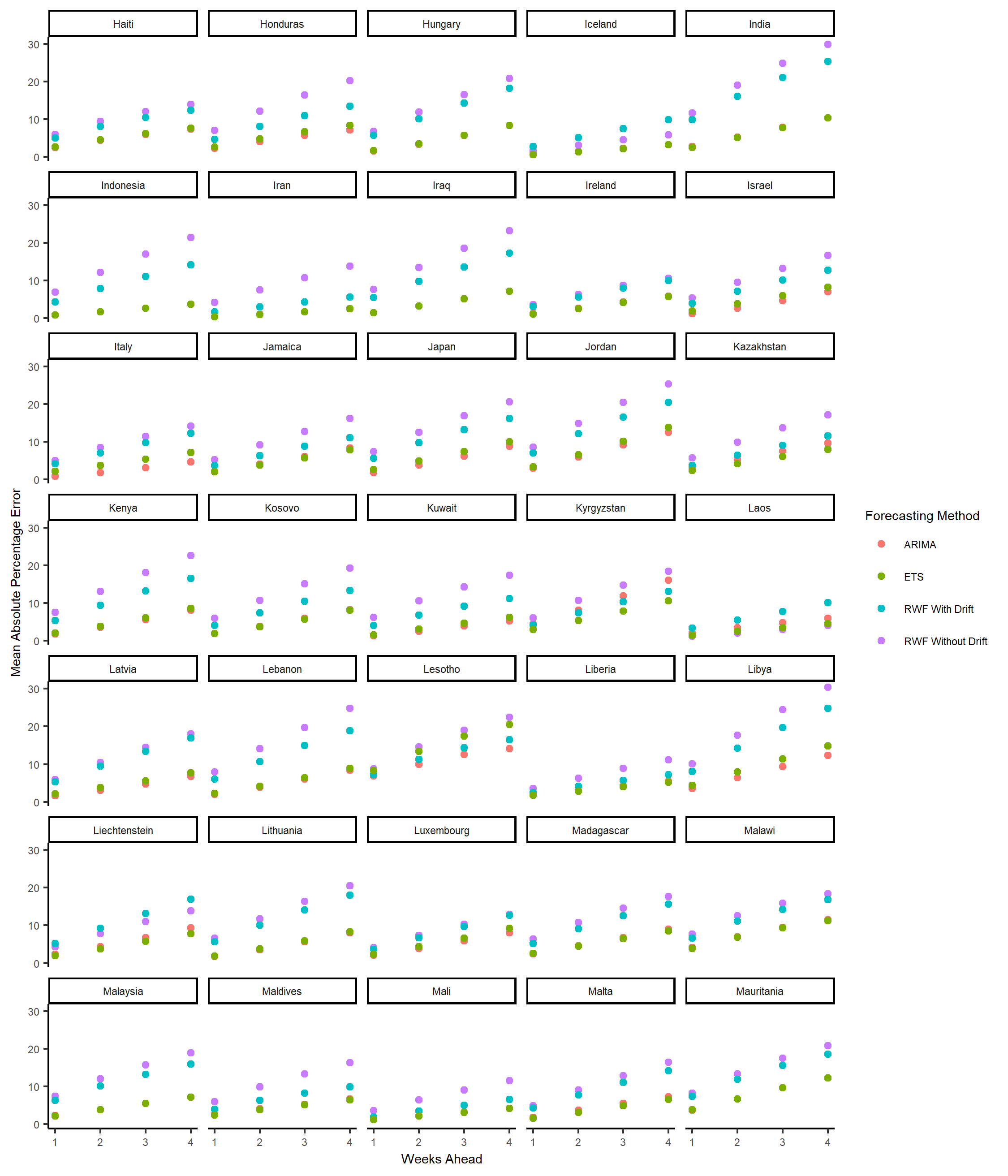

Supplement: S3 Fig — (TIF) [file pone.0252147.s007.tif]

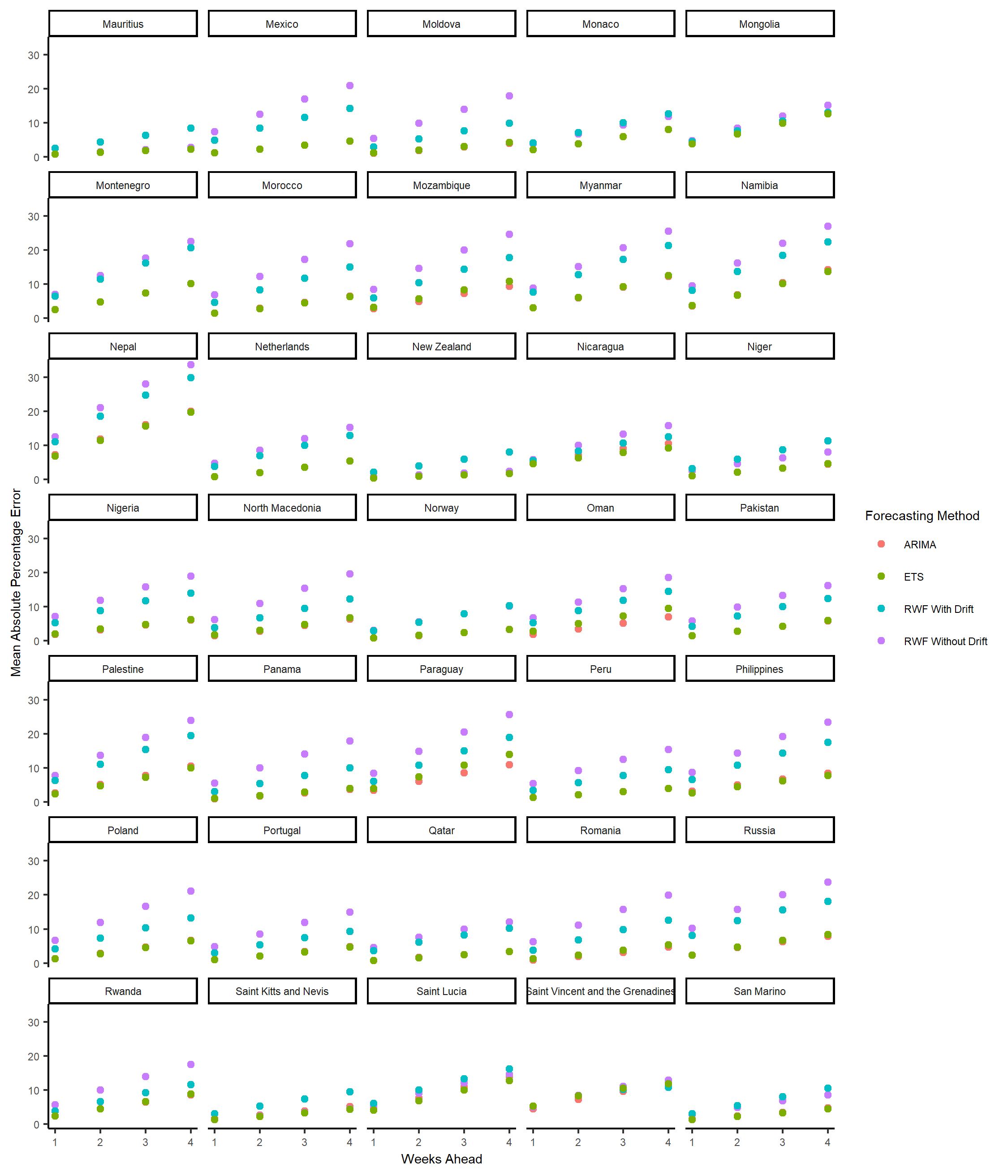

Supplement: S4 Fig — (TIF) [file pone.0252147.s008.tif]

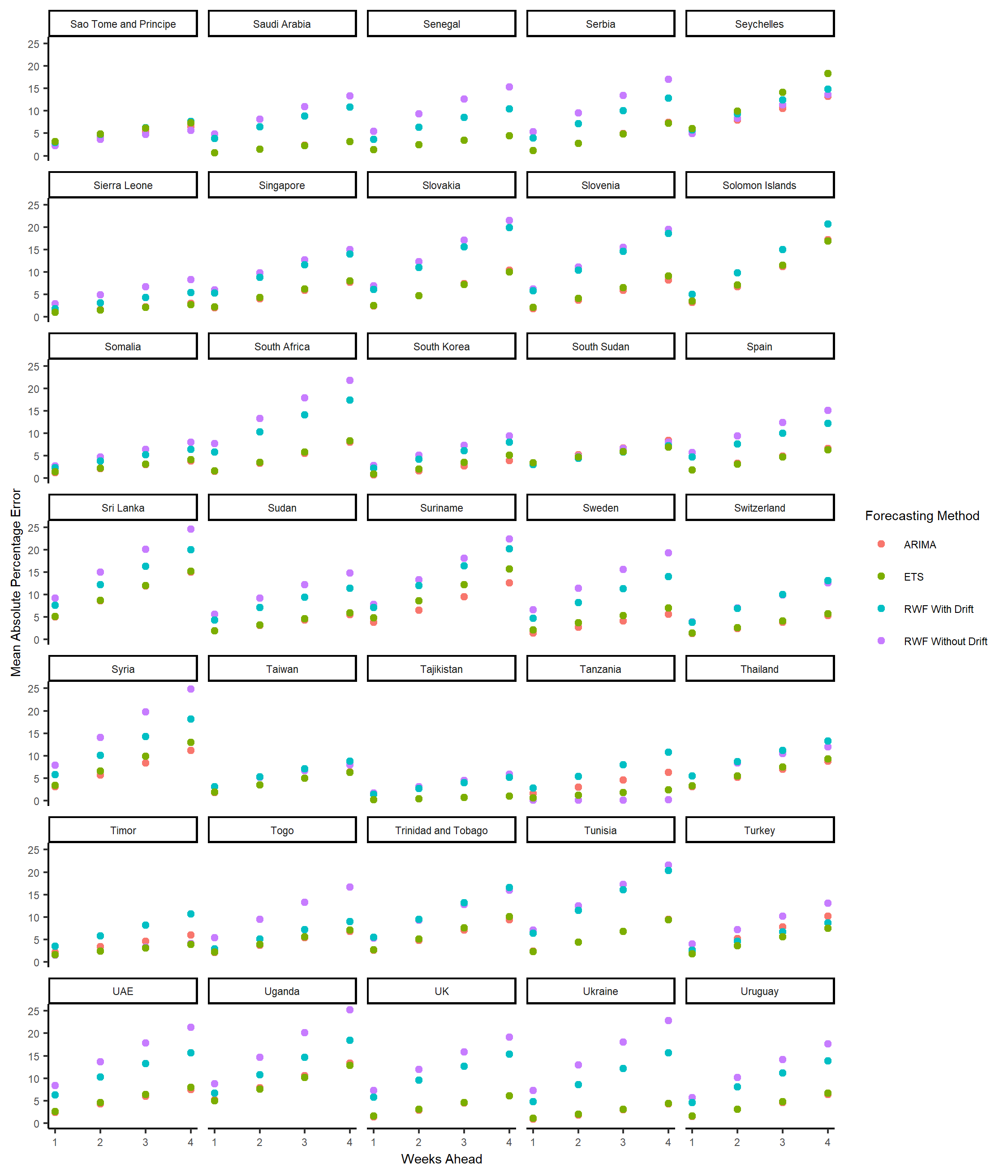

Supplement: S5 Fig — (TIF) [file pone.0252147.s009.tif]

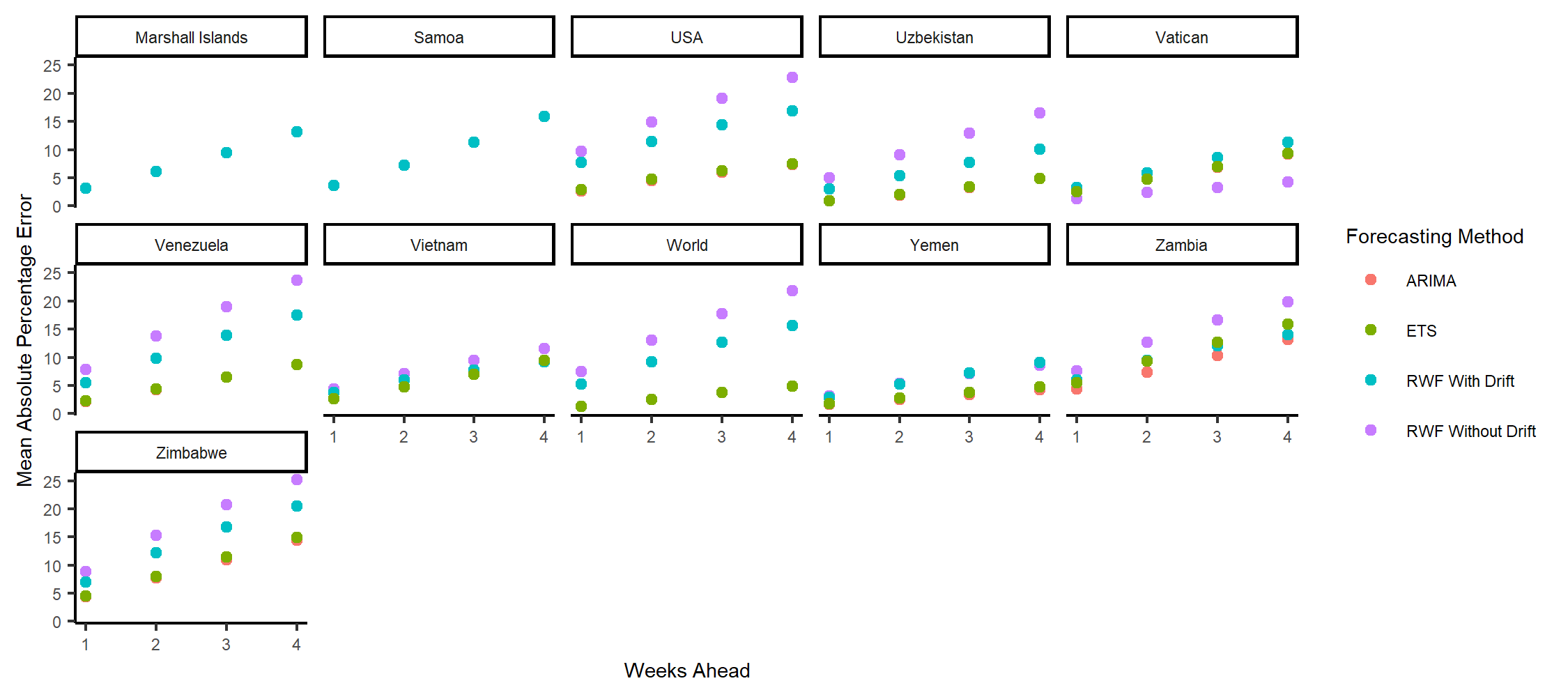

Supplement: S6 Fig — (TIF) [file pone.0252147.s010.tif]

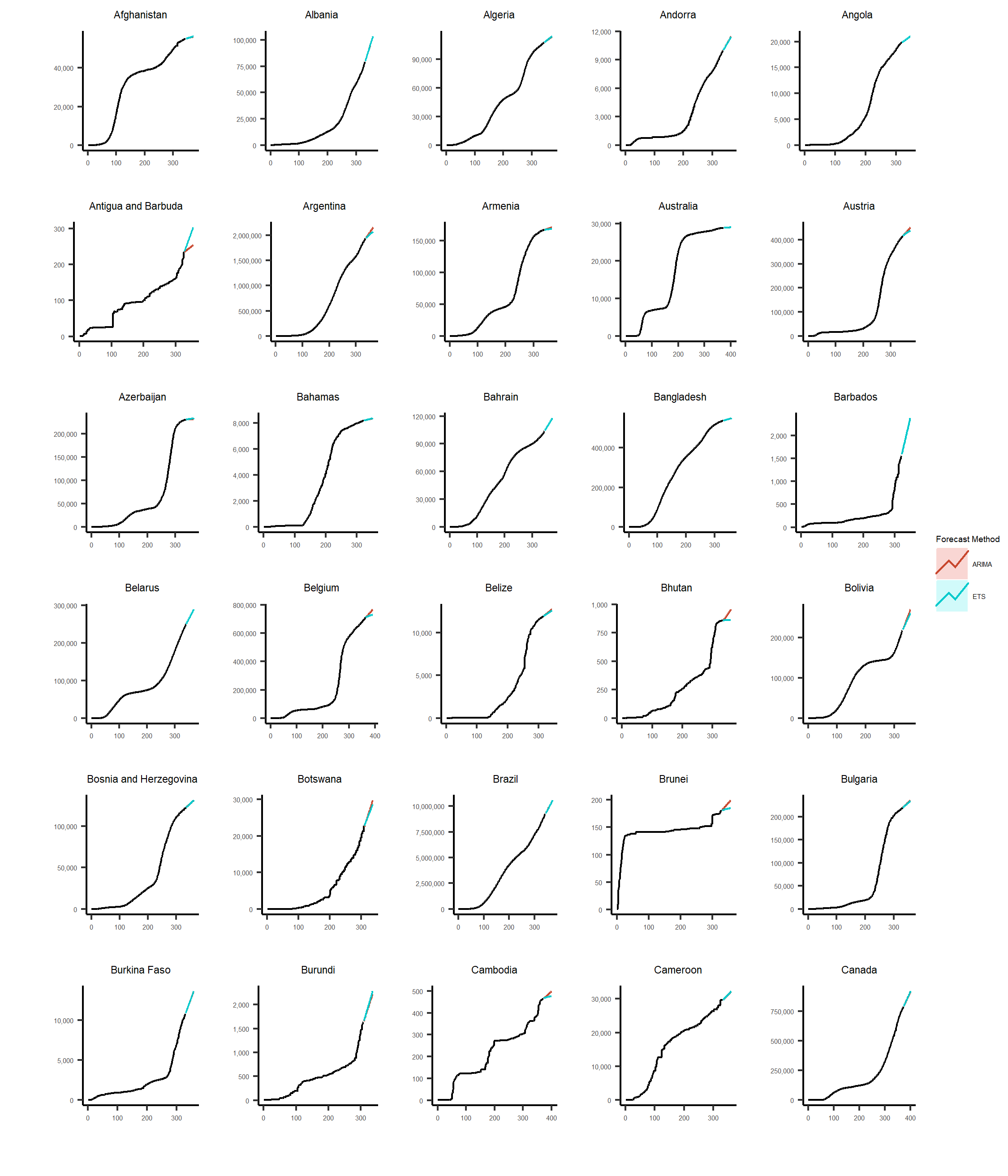

Supplement: S7 Fig — (TIF) [file pone.0252147.s011.tif]

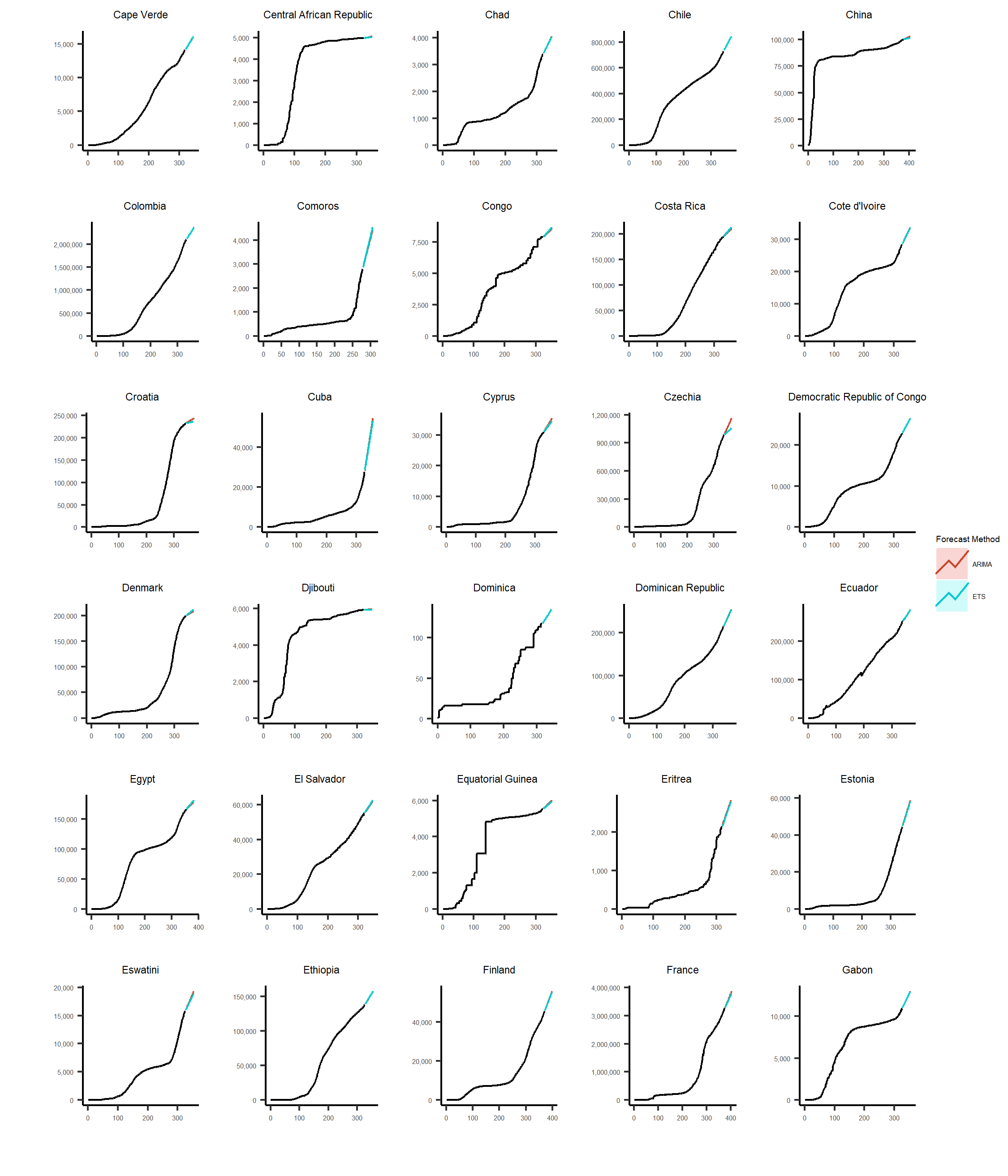

Supplement: S8 Fig — (TIF) [file pone.0252147.s012.tif]

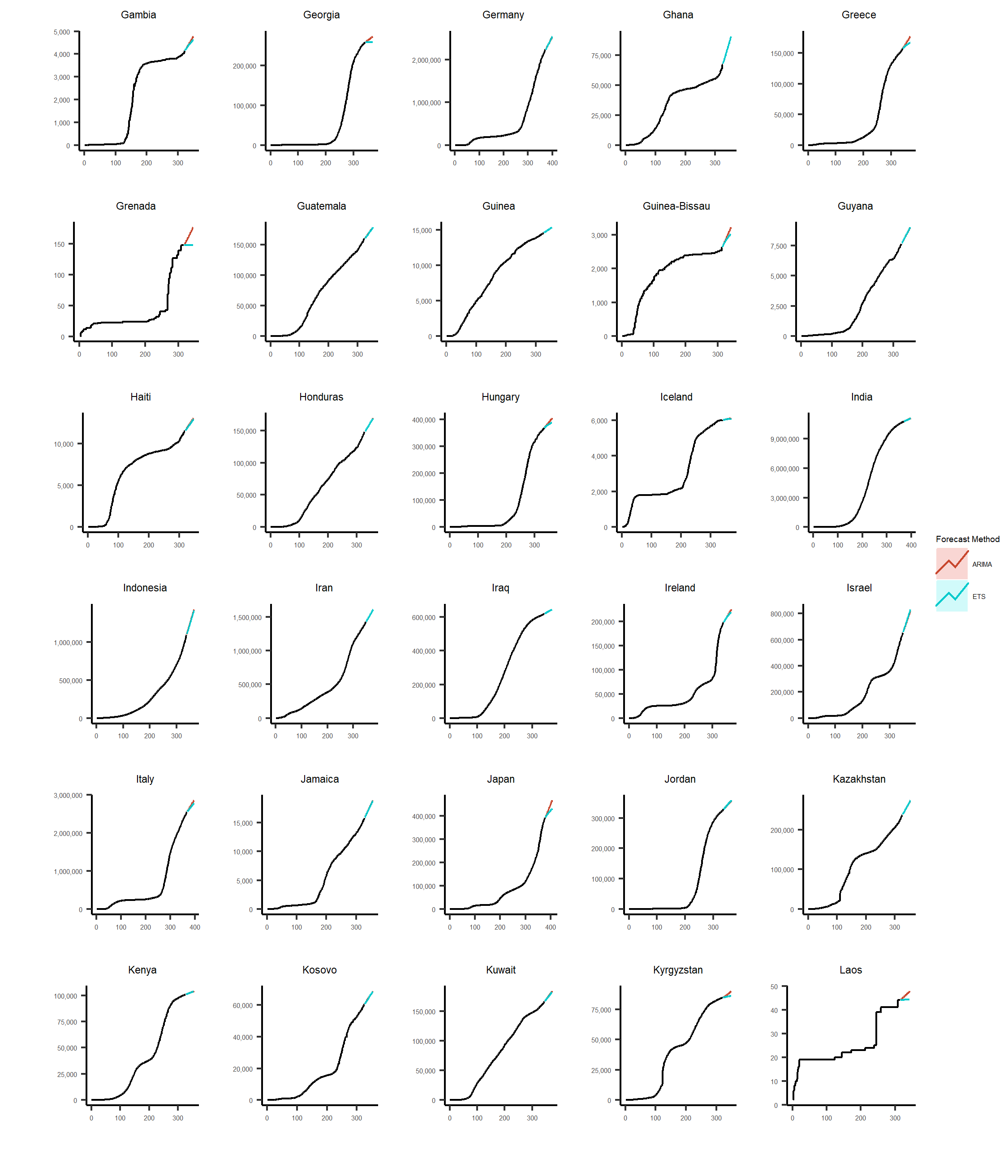

Supplement: S9 Fig — (TIF) [file pone.0252147.s013.tif]

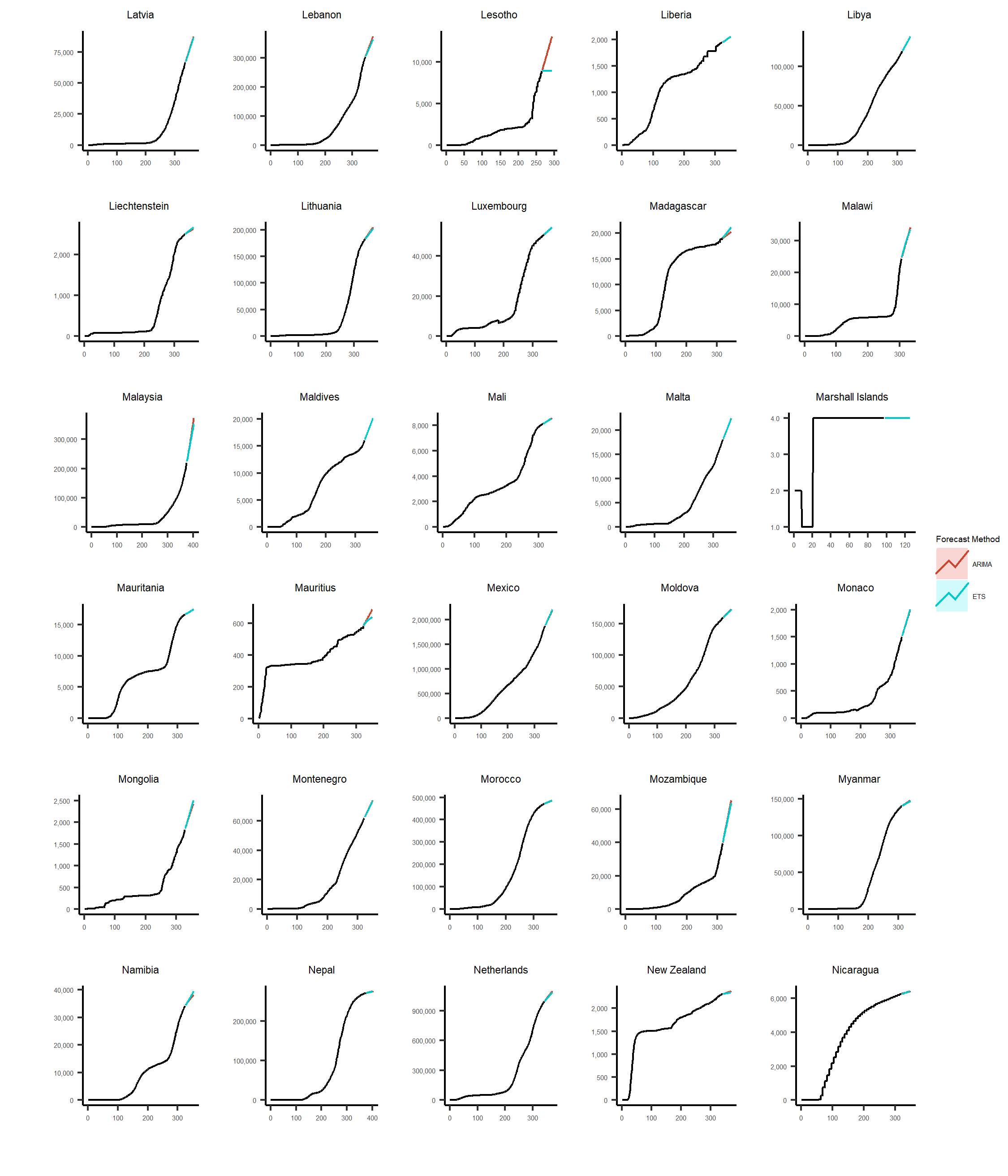

Supplement: S10 Fig — (TIF) [file pone.0252147.s014.tif]

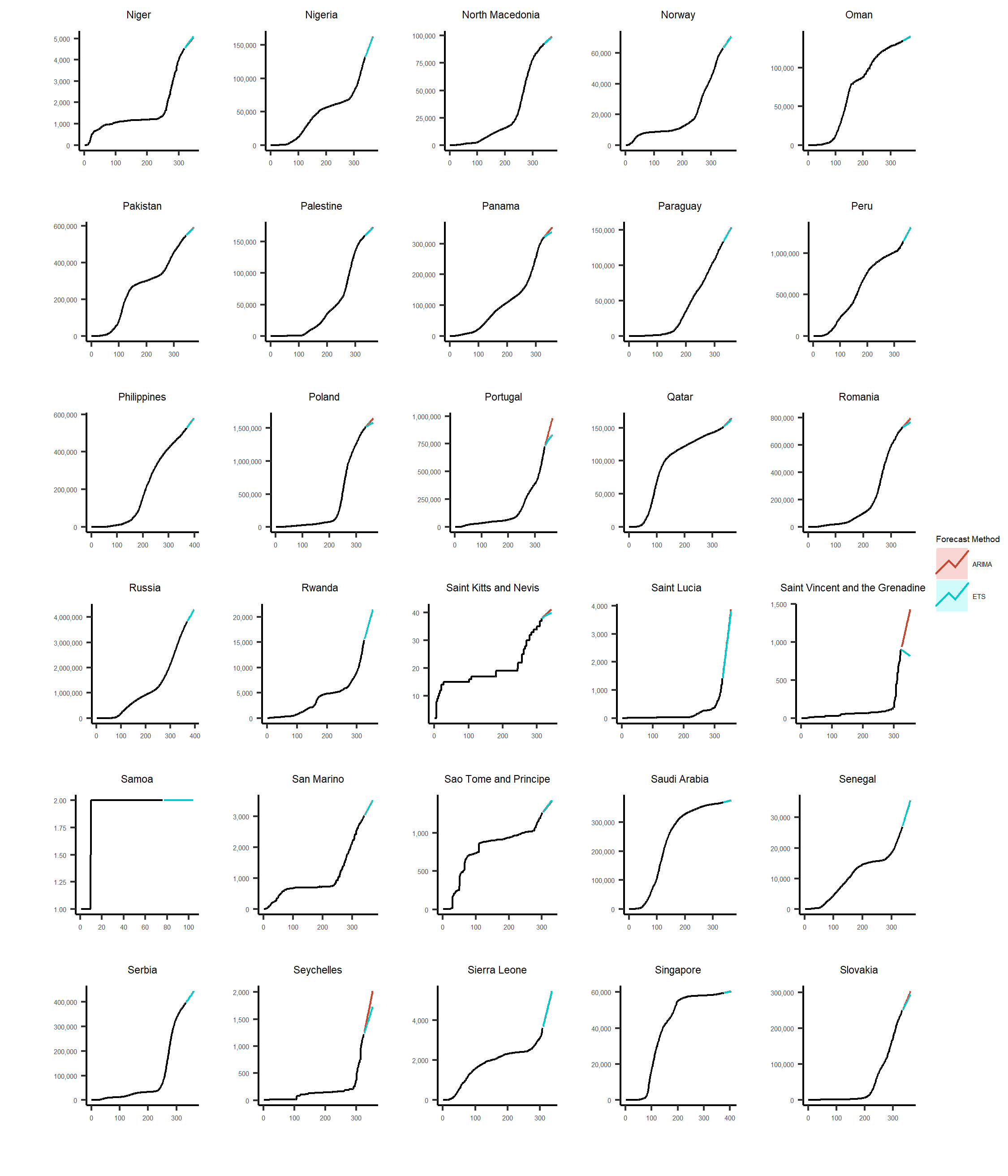

Supplement: S11 Fig — (TIF) [file pone.0252147.s015.tif]

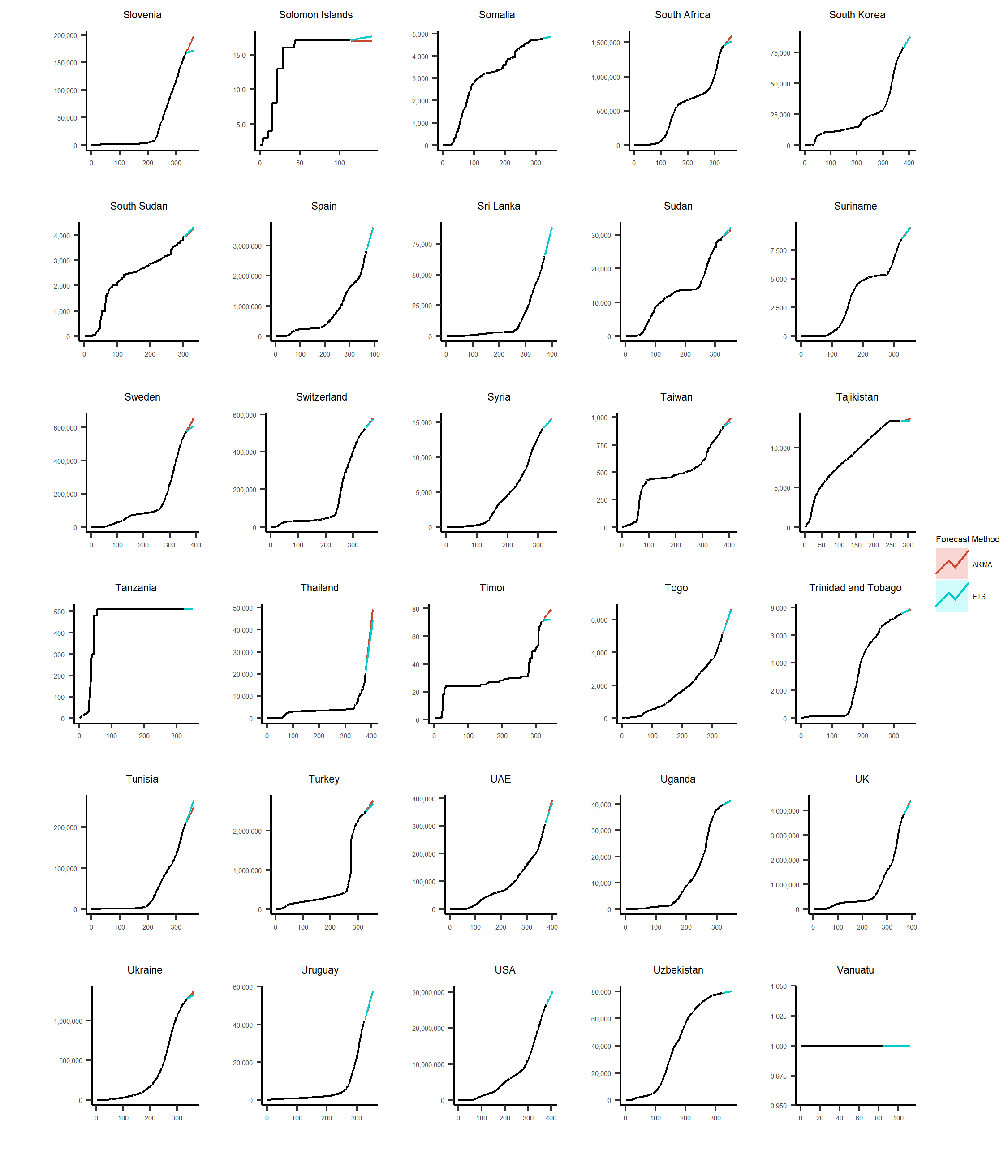

Supplement: S12 Fig — (TIF) [file pone.0252147.s016.tif]

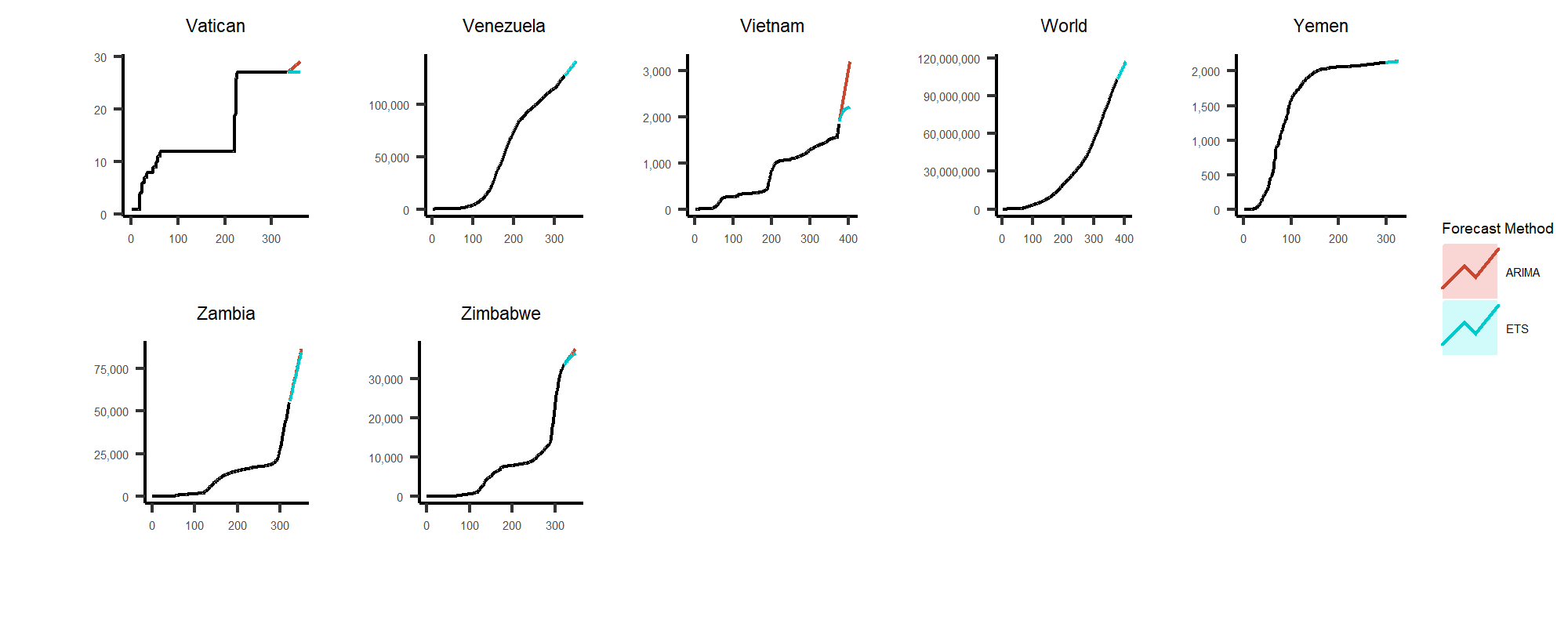

Supplement: S13 Fig — (TIF) [file pone.0252147.s017.tif]
